# Ruthenium *p*-Cymene Complexes Incorporating Substituted Pyridine–Quinoline-Based Ligands: Synthesis, Characterization, and Cytotoxic Properties

**DOI:** 10.3390/molecules29133215

**Published:** 2024-07-06

**Authors:** Afroditi Kokkosi, Elpida Garofallidou, Nikolaos Zacharopoulos, Nikolaos Tsoureas, Konstantina Diamanti, Nikolaos S. Thomaidis, Antigoni Cheilari, Christina Machalia, Evangelia Emmanouilidou, Athanassios I. Philippopoulos

**Affiliations:** 1 Laboratory of Inorganic Chemistry, Department of Chemistry, National and Kapodistrian University of Athens, Panepistimiopolis Zografou, 15771 Athens, Greece; afroditi806@gmail.com (A.K.); elpida.garofallidou@gmail.com (E.G.); nzachar@chem.uoa.gr (N.Z.); ntsoureas@chem.uoa.gr (N.T.); 2 Laboratory of Analytical Chemistry, Department of Chemistry, National and Kapodistrian University of Athens, Panepistimiopolis Zografou, 15771 Athens, Greece; kdiamanti@chem.uoa.gr (K.D.); ntho@chem.uoa.gr (N.S.T.); 3 Department of Pharmacognosy and Natural Products Chemistry, Faculty of Pharmacy, National and Kapodistrian University of Athens, Panepistimiopolis Zografou, 15771 Athens, Greece; cheilarianti@pharm.uoa.gr; 4 Laboratory of Biochemistry, Department of Chemistry, National and Kapodistrian University of Athens, Panepistimiopolis Zografou, 15771 Athens, Greece; cmachali@chem.uoa.gr (C.M.); eemman@chem.uoa.gr (E.E.)

**Keywords:** 2,2′-pyridyl-quinoline ligands, *p-*cymene, ruthenium complexes, cytotoxicity, MTT

## Abstract

Organometallic complexes of the formula [Ru(N^N)(*p*-cymene)Cl][X] (N^N = bidentate polypyridyl ligands, *p*-cymene = 1-methyl-4-(1-methylethyl)-benzene, X = counter anion), are currently studied as possible candidates for the potential treatment of cancer. Searching for new organometallic compounds with good to moderate cytotoxic activities, a series of mononuclear water-soluble ruthenium(II)–arene complexes incorporating substituted pyridine–quinoline ligands, with pending -CH_2_OH, -CO_2_H and -CO_2_Me groups in the 4-position of quinoline ring, were synthesized, for the first time, to study their possible effect to modulate the activity of the ruthenium *p*-cymene complexes. These include the [Ru(η^6^-*p*-cymene)(pqhyme)Cl][X] (X = Cl^−^ (**1-Cl**), PF_6_^−^ (**1-PF_6_**), pqhyme = 4-hydroxymethyl-2-(pyridin-2-yl)quinoline), [Ru(η^6^-*p*-cymene)(pqca)Cl][Cl] ((**2-Cl**), pqca = 4-carboxy-2-(pyridin-2-yl)quinoline), and [Ru(η^6^-*p*-cymene)(pqcame)Cl][X] (X = Cl^−^ (**3-Cl**), PF_6_^−^ (**3-PF_6_**), pqcame = 4-carboxymethyl-2-(pyridin-2-yl)quinoline) complexes, respectively. Identification of the complexes was based on multinuclear NMR and ATR-IR spectroscopic methods, elemental analysis, conductivity measurements, UV–Vis spectroscopic, and ESI-HRMS techniques. The solid-state structures of **1-PF_6_** and **3-PF_6_** have been elucidated by single-crystal X-ray diffraction revealing a three-legged piano stool geometry. This is the first time that the in vitro cytotoxic activities of these complexes are studied. These were conducted in HEK293T (human embryonic kidney cells) and HeLa cells (cervical cancer cells) via the MTT assay. The results show poor in vitro anticancer activities for the HeLa cancer cell lines and **3-Cl** proved to be the most potent (IC_50_ > 80 μΜ). In both cell lines, the cytotoxicity of the ligand precursor pqhyme is significantly higher than that of cisplatin.

## 1. Introduction

In the field of medicinal chemistry, ruthenium(II) complexes constitute an important class of anticancer agents with selective antimetastatic properties and low systemic toxicity [1,2,3,4,5]. This bioactivity could be further attributed to the reported high accumulation of ruthenium complexes in cancer cells, along with its ability to mimic iron in transport processes, and the different modes of activity observed [6]. However, according to E. Alessio, the inventor of NAMI-A (imidazolium *trans*-[tetrachloro(dimethylsulfoxide)imidazole ruthenium(III)], a potent anticancer ruthenium(III) complex, all these undemonstrated misconceptions (or myths) are in doubt, affecting the further development of this research topic [7]. In addition, it has been reported that DNA is not the only target but binding to enzymes and protein targets has already generated promising in vivo data [8]. Apparently, the pharmacological profile of the ruthenium-based drugs [9,10], differs significantly from those of the platinum analogs currently in clinical use [11,12]. The most representative examples are ruthenium(III) complexes, namely NAMI-A and KP-1019 ((indazolium *trans*-[tetrachlorobis(1*H*-indazole)ruthenium(III)]), which have successfully entered through phase I clinical trials [13,14]. Although both complexes show similar structures, they display different in vitro and in vivo activities. NAMI-A entered phase II trials but due to limited efficacy, it could not proceed further for clinical development [7]. In addition, NKP-1339, the sodium salt of KP-1019, which shows higher aqueous solubility, has entered early phase II clinical trials [15].

Within the great number of ruthenium complexes that have been investigated [16], organometallic half-sandwich complexes of the type [Ru(N^N)(*p*-cymene)Cl][X] (N^N = polypyridyl ligands and *p*-cymene = 1-methyl-4-(1-methylethyl)-benzene); X = counter anion), constitute an interesting class of compounds, which are currently studied as alternative to the classic platinum(II) analogs [17,18,19,20,21]. Representative examples of the series, among others, are the so-called RAPTA ruthenium (II) complexes, incorporating the PTA = 1,3,5-triaza-7-phosphatricyclo-[3.3.1.1]decane (termed as RAPTA) ligand, to increase water solubility and subsequently making ruthenium–chlorine bonds prone to hydrolysis [22]. Despite the rather low in vitro cytotoxicity of RAPTA-C, a representative example of the series, this complex shows selectivity towards tumors in vivo and has attracted significant attention for the development of new anticancer agents [23]. Generally, RAPTA complexes have shown higher affinities for protein binding over DNA [24]. Early studies by Sadler et al. and Dyson et al., pioneers in the field, trace back to early 2010 [25,26]. These studies have led to structure–activity relationships, which among others suggest the importance of Cl ligand as an easily leaving group along with the role of the ancillary bidentate ligand for regulation of reactivity against DNA binding and/or proteins. In addition, the overall charge of the complex is of importance, since it determines the solubility and possible permeability of the complex [27]. Also, it has been reported that the spectator η^6^-arene ligand under physiological conditions, plays a crucial role since not only stabilizes the ruthenium(II) center but also provides the hydrophobic character required, while the ruthenium remains the hydrophilic center. Several organometallic half-sandwich ruthenium(II) complexes have been prepared and tested as possible candidates for the potential treatment of cancer disease [28,29,30,31,32,33,34]. Two recent review articles highlight the current progress on the ruthenium(II)–arene complexes along with possible mechanistic pathways [35,36]. 

As a continuation of our ongoing research interest in the field, we report herein the synthesis, spectroscopic and structural characterization of a series of mononuclear ruthenium(II) complexes of the general formula, [Ru(η^6^-*p*-cymene)(pqhyme)Cl][X] (X = Cl^−^ (**1-Cl)**; PF_6_^−^ (**1-PF_6_**); pqhyme = 4-hydroxymethyl-2-(pyridin-2-yl)quinoline), [Ru(η^6^-*p*-cymene)(pqca)Cl][Cl] (**2-Cl**; pqca = 4-carboxy-2-(pyridin-2-yl)quinoline), and [Ru(η^6^-*p*-cymene)(pqcame)Cl][X] (X = Cl^−^ (**3-Cl**); PF_6_^−^ (**3-PF_6_**), pqcame = 4-carboxymethyl-2-(pyridin-2-yl)quinoline). Considering our previous experience with pyridine–quinoline ligands bearing -COOH, and -COOMe pending moieties, we wanted to synthesize new ruthenium complexes with –CH_2_OH as well. The ligands studied belong to the class of substituted pyridine–quinoline with pending -CH_2_OH, -CO_2_H, and -CO_2_Me groups in the 4-position of quinoline ring, whose coordination chemistry has been examined to a small extent. Moreover, and since it is well known that the quinoline moiety participates as a scaffold in numerous biological activities [37], we wanted also to study the possible effect of the side groups, to modulate the biological potencies of the ruthenium *p*-cymene complexes. To the best of our knowledge, there are no biological reports for the relevant pyridine–quinoline ligands thus far. Surprisingly, also there are no biological reports (cytotoxic studies, etc.), regarding the ruthenium(II)-*p*-cymene analogs mentioned above. 

In this context, initial attempts were undertaken to study and evaluate the in vitro cytotoxic properties of the relevant substances (ligand precursors and metal complexes, except for **2**) in healthy HEK293T (human embryonic kidney cells) and HeLa cells (cervical cancer cells), via the MTT assay. 

## 2. Results and Discussion

### 2.1. Chemistry

**Scheme 1:**
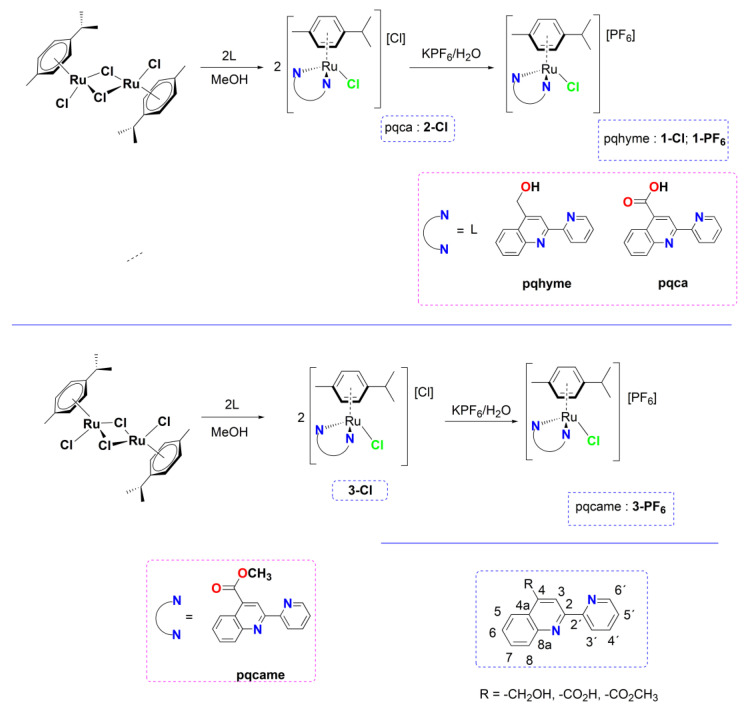
Reaction scheme and conditions for the synthesis of ruhenium(II) complexes reported. Atom numbering for NMR assignment is also included.

The present work focuses on the synthesis and spectroscopic characterization of new ruthenium(II) half-sandwich organometallic complexes of the general type [Ru(η^6^-*p*-cymene)(L)Cl][X] (X = Cl^−^, PF_6_^−^) and the study of their cytotoxicity properties. To achieve this, substituted pyridyl-quinoline ligands tethered in the 4-position of quinoline with -CH_2_OH (pqhyme), -CO_2_H (pqca), or -CO_2_Me (qcame) groups, were selectively chosen (Scheme 1). Notably, from a literature survey performed we have realized that the organometallic chemistry of those ligands has not been described thus far. 

The chlorido ruthenium(II) complexes **1-Cl, 2-Cl,** and **3-Cl** were prepared in a one-pot reaction, upon treatment of the dinuclear precursor [Ru(η^6^-*p*-cymene)(μ-Cl)Cl]_2_, with two mol equivalents of the corresponding ligand (pqhyme, pqca, and qcame) in dry methanol. The general synthetic route and reaction conditions are presented in Scheme 1. Interestingly, we also noticed that the coordination chemistry of the pqhyme ligand has not been examined in detail [38].

Emphasis is given to the characterization of the chloride salts that are more appropriate for biological studies (solubility in water). Their identity was established by a combination of FT-IR, multinuclear NMR spectroscopy, satisfactory elemental analyses, mass spectrometric analysis along with UV–Vis spectroscopy, and molar conductivity measurements. The relevant complexes **1-Cl** and **3-Cl** were transformed to the corresponding PF_6_^−^ analogs, which generally can provide higher quality single crystals as compared to the Cl^−^ congeners (vide infra in X-ray section). For **2-Cl** however, that contains the acidic -COOH moiety, treatment with a saturated KPF_6_(aq) solution afforded the neutral complex **2**, where the PF_6_^−^ counter ion is absent, and the carboxylic acid group has been deprotonated (vide infra). 

The mononuclear organometallic complex **1-Cl** comprising the pqhyme ligand was isolated as an orange–brown solid in high yield. It dissolves in DMSO, and water and is less soluble in CHCl_3_. Subsequent treatment with a saturated aqueous KPF_6_ solution afforded, via a metathesis reaction, the hexafluorido analog **1-PF_6_**. Both complexes are air-stable in the solid state and melt with decomposition at 155 °C and 137 °C, respectively. The FT-IR spectra (ATR mode) of both complexes are almost identical, as expected for complexes that display very similar structures. For **1-Cl** the presence of water of crystallization is evident from the intense and broad bands at ∼3350–3250 cm^−1^ due to the antisymmetric and symmetric ν(O-H) stretching vibration modes [39]. The O–H stretching vibration band from pending -CH_2_OH moiety is hampered within the broadness of the band reported above. Also, the strong band at approximately 1650 cm^−1^ is due to the δ(H–O-H) bending mode. The characteristic bands at 3061 cm^−1^, and in the region of 2998–2850 cm^−1^ correspond to ν(C–H) aromatic and aliphatic stretching vibrations modes of the ligand, while the medium intensity band at ∼800 cm^−1^ is typical for the stretching vibration mode of ν(Ru–C) [40]. In the region of 800-600 cm^−1^, the spectrum is dominated by the very strong in-plane and out-of-plane deformation bands from the pyridine ring of pqhyme ligand. For **1-PF_6,_** the two very strong bands at 840 cm^−1^ and 557 cm^−1^ are assigned to the ν_3_(P–F) and ν_4_(P–F) vibration modes of the PF_6_^−^ anion (Figure S1 of the Supplementary Materials) [41].

The solution NMR spectra (^1^H and ^13^C{^1^H}) of **1-Cl** and **1-PF_6_** in CDCl_3_ feature the characteristic set of resonances of the pqhyme and p-cymene ligands supporting the proposed formulae of the compounds (Figures S2–S5 of the Supplementary Materials) [42]. Atom numbering for NMR assignment is included in Scheme 1. The resonance signals attributed to the aromatic ring protons of pqhyme are shifted downfield in comparison to the free ligand, furthermore suggesting coordination to the ruthenium(II) center. The separate and well-resolved resonance signals at δ 5.92–5.61 in a ratio of 1:1:1:1 (according to integration) are typical of the p-cymene ring protons. The characteristic singlet resonance signals at δ 5.47 for **1-Cl** and 5.48 for **1-PF_6_** are attributed to the -CH_2_ protons of the pending -CH_2_OH moiety. These were upfield shifted compared to the *p*-cymene ligand of the metal precursor [43]. In addition, the septet resonance signals at δ 2.29 for **1-Cl** and δ 2.44 for **1-PF_6_** are indicative of the -CH(CH_3_)_2_ proton, while the two doublets of doublets at δ 0.87/0.91 (**1-Cl**) and δ 0.93/0.97 (**1-PF_6_**) can be assigned to the methyl protons of the isopropyl group. Proton assignment for **1-PF_6_** was facilitated with the help of a ^1^H–^1^H COSY spectrum as shown in Figure S6. For **1-Cl**, and after two days of standing in CDCl_3_, free *p*-cymene was detected (c.a. 2.6%) along with the formation of new unknown species. The ^1^H NMR pattern of the spectrum remained practically unchanged (Figure S7). 

Initial attempts to obtain single crystals of **1-Cl** failed. However, upon the reaction of [Ru(η^6^-*p*-cymene)(μ-Cl)Cl]_2_ and pqhyme in a 1:4 metal-to-ligand molar ratio, we were able to obtain clear yellow plates, from a concentrated solution of the complex in CDCl_3_. The solid-state structure corresponds to the formula **1-Cl**•2CHCl_3_•pqhyme suggesting that the excess of pqhyme ligand has been co-crystallized, including two CHCl_3_ molecules [42]. The geometry of this complex can be described as distorted octahedral coordination, with the *p*-cymene ligand coordinated in a η^6^-fashion to the ruthenium center. The structure was refined to an R final index of 0.12, thus the exact determination of the bond distances and bond angles are not sufficiently accurate. In any case, the structural data are very useful for comparison to other similar ruthenium complexes and not only to show connectivity (Figure S8 and Table S1 of the Supplementary Materials). In the unit cell, the co-crystallized pqhyme ligand adopts a cis orientation (Figure S9), which can be attributed to crystal packing forces and a classic hydrogen bonding interaction between the counter anion and oxygen atom O(2) from the co-crystallized organic ligand (O(2)-H(2)**∙∙∙**Cl(2′) = 3.035 Å, bond angle = 176.35°. Further stabilization within the crystal is provided by the intermolecular hydrogen bonding O(1)-H(1)**∙∙∙**Cl(2) = 3.099 Å; bond angle = 160.7° and O(1′)-H(1′)**∙∙∙**Cl(2′) = 3.051 Å; bond angle = 158.6°. A list of hydrogen bonding interactions within the crystal is included in the Supplementary Material Table S2.

**Figure 1:**
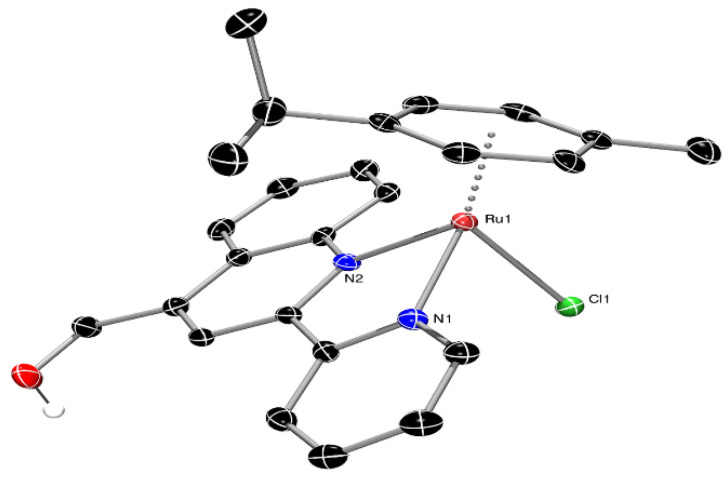
ORTEP diagram of the molecular structure of complex cation [Ru(η^6^-p-cymene)(pqhyme)Cl]^+^ in the solid state. Thermal ellipsoids are set at 50% probability. Hydrogen atoms and the PF_6_^−^ counter anion are omitted for clarity. Selected bond lengths (Å) and angles (°) for the cation of **1-PF_6_**: Ru-(cym) ring centroid = 1.6900(3), Ru-Cl = 2.4088(10), Ru-N(1) = 2.088(3), Ru-N(2) = 2.126(3); N(2)-Ru-N(1) = 76.74(11).

Subsequently, we managed to obtain high-quality single crystals of the relevant hexafluorido derivative **1-PF_6_**. The solid-state structure of **1-PF_6_** was determined by single-crystal X-ray diffraction studies. Suitable yellow blocks were obtained upon the slow evaporation of diethyl ether into a methanol solution of this complex, at ambient temperature. The molecular structure of the complex is depicted in Figure 1 and selected bond lengths and angles with estimated standard deviations, are included in the legend of the Figure. Complex **1-PF_6_** crystallizes in the monoclinic crystal system and space group P2_1/c_. The geometry of this complex can be described as three-legged piano stool, with the ruthenium atom coordinated in an η^6^-fashion to the p-cymene ligand. The remaining basal sites are occupied by a chlorine atom and two N atoms of the chelating ligand pqhyme. The Ru–Cl bond length of 2.409 Å and the Ru–N1 and Ru–N2 bond lengths of 2.088 Å and 2.126 Å, respectively, are in the same range as other similar ruthenium(II) complexes [44]. Also, the ruthenium to p-cymene ring centroid bond distance of 1.690 Å, complies with the relevant values reported for analogous complexes [45]. The planes defined by the pyridine (N1–C46–C50) and quinoline rings (N2–C38–C45) deviate from planarity by 11.83^o^. Deviation from the ideal octahedral coordination geometry is provided by the N(2)–Ru–N(1), N(1)–Ru–Cl, and N(1)–Ru–Cl bond angles of 76.74°, 85.61°, and 86.92°, respectively. Finally, the crystal of **1-PF_6_** is stabilized by intermolecular non-classical C–H∙∙∙π contacts, [distance of (C7A–H7A)∙∙∙centroid (C1–C6) = 3.366 Å; distance of (C9C–H9C)∙∙∙centroid (C15–C19) = 3.793 Å, Figure S10).

The [Ru(η^6^-p-cymene)(pqca)Cl]Cl complex **2-Cl** was isolated in 85% yield as an orange–red air-stable solid that melts at 125 °C. Upon further heating, decomposition occurs at 150 °C, accompanied by gas evolution, leading to a black solid [46]. It is soluble in polar organic solvents (MeOH, EtOH, water) including DMSO and DMF.

The cationic complex **2-Cl** displays a strong absorption for the ν_as_(C=O) stretching vibration at 1704 cm^−1^, while the ν(C–O) mode is present as a characteristic band at 1200 cm^−1^. This further implies that the COOH group remains intact and is not involved in coordination with the ruthenium(II) center. Bands in the region of 1549–1152 cm^−1^ can be tentatively assigned to ν(C=C) and ν(C=N) vibration modes, respectively, while the intense absorption bands below 800 cm^−1^ are typical for in-plane and out-of-plane deformation bands from the pyridine ring of pqca ligand (Figure S11).

The ^1^H NMR spectrum of **2-Cl** was recorded in DMSO-d_6_ at ambient temperature. The aromatic region of the spectrum of this complex displays sharp and well-resolved resonance signals attributable to the pyridyl and quinoline protons of the pqca ligand, while the resonance signal of the carboxylic acid proton was not detected under these conditions. In this solvent, complex **2-Cl** practically remains stable as was evidenced by recording its ^1^H NMR spectrum over time. The stability of **2-Cl** in DMSO is shown below in Section 2.1.1. The aromatic region of the spectrum of complex **2-Cl** displays nine sharp (four doublets and one singlet) and well-resolved resonance signals. The singlet resonance at δ 8.94 is typical for H3 of the bidentate ligand, while the four separate doublet resonance signals in between δ 6.19, 6.13, 6.03, and 5.97 correspond to the p-cymene ring protons. In addition, the high field region of the ^1^H NMR spectrum of **2-Cl** shows a septet signal at δ 2.25 ppm for the -CH-(CH_3_)_2_ protons of the p-cymene ligand that is overlapped with the intense singlet resonance at δ 2.27 for the -CH_3_ group of p-cymene. In this region, the CH_3_ protons of the -CH-(CH_3_)_2_ moiety appear as two doublet resonances at δ 0.81 and 0.77, respectively. The ^13^C NMR signals of **2-Cl** were assigned by two-dimensional ^1^H–^13^C HSQC and ^1^H–^13^C HMBC methods and compared with the literature data from similar complexes [47]. In the aromatic region, the ^13^C{^1^H} NMR spectrum of the complex shows the expected number of characteristic resonance signals corresponding to the pyridyl and quinoline ring carbons including also the relevant signals from the carbon atoms of the p-cymene ring atoms. Analyzing the spectrum, the resonance signal at δ 166.31 is assigned to the carbon atom of the carboxylic acid group of pqca. In the aliphatic region of the spectrum, the carbon atoms from the CH_3_ groups of p-cymene ligand appear as intense singlets at δ 21.58, 21.21, and 18.18, respectively (Figures S12–S15).

The [Ru(η^6^-*p*-cymene)(pqcame)Cl]Cl analog **3-Cl** was isolated almost quantitatively as a dark brown air-stable solid that decomposes at 111 °C. It dissolves in water, DMSO, and other polar solvents. The FT-IR spectrum of **3-Cl** is characterized by a strong asymmetric stretching vibration typical of the ν_as_(C=O) of the ester moiety at 1722 cm^−1^, shifted to higher wave numbers compared with **2-Cl**. Subsequent treatment of **3-Cl** with a saturated aqueous KPF_6_ solution resulted in **3-PF_6_** in 75% yield. This is an air-stable orange solid that decomposes upon heating at 190 °C. 

The FT-IR spectra of both complexes are quite similar except for the two very strong vibration bands at 825 cm^−1^ and 555 cm^−1^, attributed to the vibration modes of the PF_6_^−^ counter anion (Figure S16). Finally, the assignment of the low to medium intensity stretching vibration mode of ν(Ru-C), at 799 cm^−1^ and 798 cm^−1^, respectively, became apparent, upon a careful comparison of the IR spectra of both complexes with that of the dinuclear precursor [Ru(η^6^-*p*-cymene)(μ-Cl)Cl]_2_ [48] and other similar ruthenium(II)–arene complexes [45].

The ^1^H and ^13^C NMR spectra of **3-Cl** and **3-PF_6_** including two-dimensional ^1^H–^1^H COSY and /or ^1^H–^13^C HSQC methods are provided in the Supplementary Materials (Figures S17–S23). The singlet resonance signal at δ 4.15 **(3-Cl)** and δ 4.14 for (**3-PF_6_**) is characteristic and can be assigned to the methyl protons of the -CO_2_Me group. In the ^13^C{^1^H} NMR spectra of both complexes, the methyl carbon of the -CO_2_Me group appears at δ 53.90 (**3-Cl**) and δ 54.13 (**3-PF_6_**), respectively.

**Figure 2:**
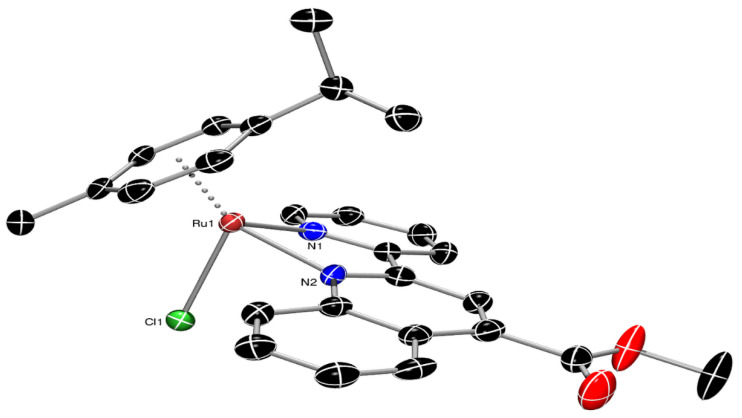
ORTEP diagram of the molecular structure of complex cation [Ru(η^6^-*p*-cymene)(pqcame)Cl]^+^ in the solid-state. Thermal ellipsoids are set at 50% probability. Hydrogen atoms and the PF_6_^−^ counter anion are omitted for clarity. Selected bond lengths (Å) and angles (°) for the cation of **3-PF_6_** in the asymmetric unit: Ru-(C-C) ring centroid = 1.7002(3), Ru-Cl = 2.3883(10), Ru-N(2) = 2.129(3), Ru-N(1) = 2.075(3); N2-Ru1-N1: 76.42(13).

Complex **3-PF_6_** crystallizes in the triclinic crystal system and space group P-1, adopting the expected pseudo-octahedral coordination geometry. The molecular structure of the complex including selected bond lengths and angles is presented in Figure 2. The p-cymene ligand is coordinated in an η^6^-fashion to the ruthenium center while the other coordination sites are occupied by one chloride ligand and two nitrogen atoms from the bidentate pqcame ligand. The planes defined by the pyridine (N1–C20–C24) and quinoline rings (N2–C11–C19) deviate from planarity by 14.28°. The overall structure characteristics (bond lengths and angles) of the complex practically do not differ from those of **1-PF_6_** and are comparable to those of analogous ruthenium(II) complexes with N-containing bidentate ligands [49,50]. Ongoing from **1-PF_6_** to **3-PF_6,_** the corresponding ruthenium(II)–cymene (ring centroid) bond distance remains intact, within the range of ∼1.70 Å, showing that relevant pending group (-CH_2_OH, -CO_2_Me) in the 4-position of the ligand, has no influence on that. For **3-PF_6_**, stabilization in the crystal is provided by intramolecular and intermolecular non-classical C–H∙∙∙π contacts, [distance of (C36B–H36B)∙∙∙centroid (C1–C5) = 4.459 Å; bond angle = 143.14°, distance of (C19B–H19B)∙∙∙centroid (C21–N4–C29) = 2.895 Å; bond angle = 139.05°, Figure S24) along with non-classical intramolecular hydrogen bonding interactions, respectively (distance of O3⋯H19C–C19C = 2.484 Å; bond angle = 168.21°).

#### 2.1.1. Solution Behavior (UV–Vis Spectroscopy, Conductivity Measurements, and Electrospray Ionization—High-Resolution Mass Spectrometry (ESI-HRMS))

**Figure 3:**
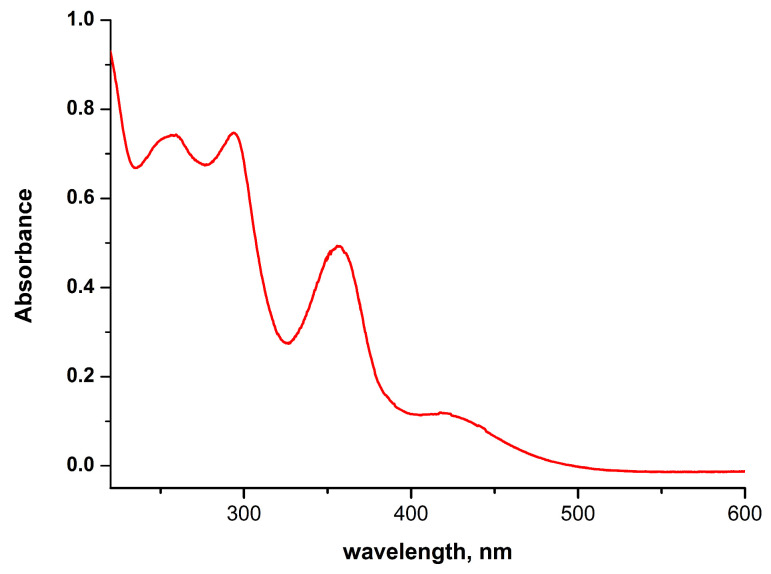
The UV–Vis spectrum of **3-Cl** in H_2_O (7.7 × 10^−5^ M).

To ensure the stability of the complexes for the in vitro cytotoxicity measurements, UV–Vis and ^1^H NMR spectroscopic techniques were undertaken. UV–Vis spectra were recorded in DMSO (not dry), for complexes **1-Cl** and **2-Cl** (Figures S25–S27) and in water for complex **3-Cl** (Figure 3), at ambient temperature, immediately after dissolution. The absorption spectra of **1-Cl** and **2-Cl** show a broad absorption band centered at ~435 nm, as reported for other ruthenium(II) half-sandwich arene complexes [51]. For **3-Cl**, this band becomes weaker and is blue-shifted to 422 nm. Ongoing from **1-Cl** to **3-Cl**, the set of high energy bands observed at 354 nm/296 nm (**1-Cl**), 357 nm/298 nm (**2-Cl**), and 357 nm/293 nm (**3-Cl**), are typical of metal-to-ligand charge transfer absorption bands (MLCT) and ligand-centered π–π* transitions. Within the series, ε values for these transitions range from 3000 to 9600 dm^3^ mol^−1^ cm^−1^, with the methyl ester analog **3-Cl**, displaying the higher extinction coefficient value of ∼9600 dm^3^ mol^−1^ cm^−1^. 

To investigate further the solution chemistry of the relevant complexes, molar conductance measurements were performed in various solvents based on their solubility potencies. The Λ values of **1-Cl, 1-PF_6,_** and **2-Cl**, upon dissolution in DMSO, were 21, 22, and 47 S cm^2^ mol^−1^, respectively [52]. Values of molar conductivity for **3-Cl** (in H_2_O) and **3-PF_6_** (in acetone) were 100 S cm^2^ mol^−1^, and 103 S cm^2^ mol^−1^, respectively. These molar conductivity data clearly indicate the ionic character of these complexes, consistent with a 1:1 electrolyte in these media [53]. 

**Figure 4:**
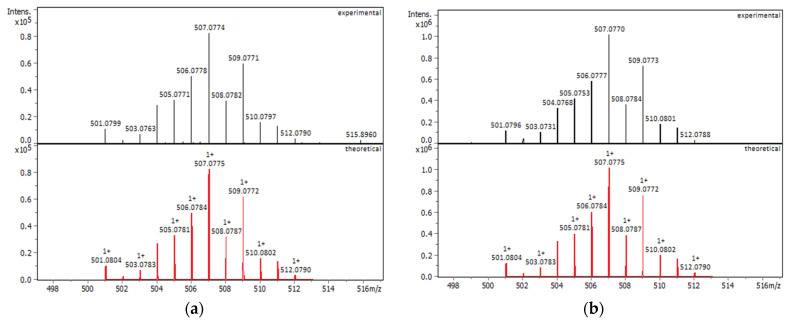
(**a**) ESI-QTOF-MS spectrum of **1-Cl** in methanol (theoretical spectrum in red line); (**b**) ESI-QTOF-MS spectrum of **1-PF_6_** in methanol (theoretical spectrum in red line).

Additional proof of the nature and the purity of all complexes examined is provided by electrospray ionization high-resolution mass spectrometry (ESI-HRMS). All complexes were dissolved in methanol reaching the appropriate concentration required and measured in the positive mode. The observed *m*/*z* values were within the acceptable threshold (±5 mDa) compared to the theoretical *m*/*z* values with good isotopic pattern fitting. For the mononuclear complexes **1-Cl** and **1-PF_6_**, the ESI-HRMS spectra present the most abundant peaks at m/z 507.0774 and 507.0770, respectively, and the isotopic patterns shown in Figure 4, which correspond to the gaseous monocation [C_25_H_26_ClN_2_ORu]^+^.

**Figure 5:**
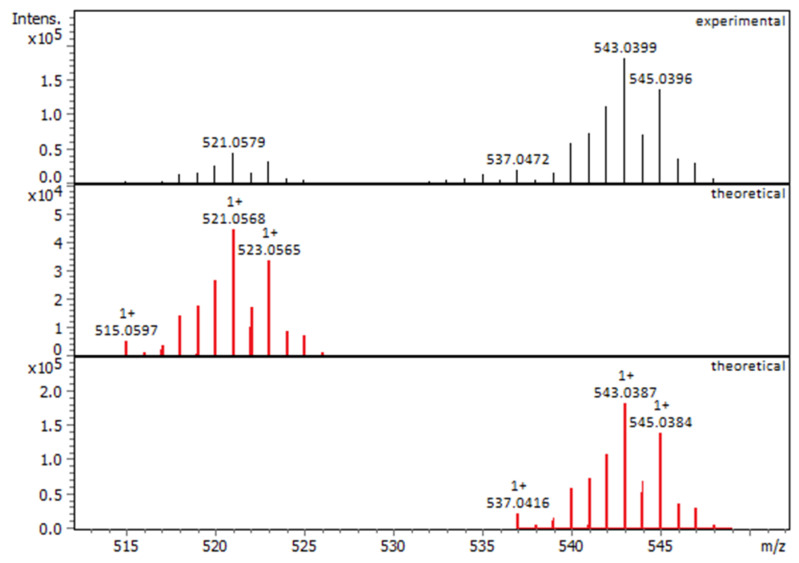
ESI-QTOF-MS spectrum of **2-Cl** in methanol (theoretical spectrum in red line).

The molecular composition of **2-Cl** was confirmed by HRMS data, exhibiting peak envelopes at *m/z* 521.0579 (40% relative abundance) and *m/z* 543.0399 (100% relative abundance), which correspond to the most abundant isotopes of [C_25_H_24_ClN_2_O_2_Ru]^+^ with mass error at 1.1mDa and the sodium adduct ion [C_25_H_23_ClN_2_O_2_RuNa]^+^ with mass error at 1.2 mDa, respectively. Good isotopic pattern fitting was observed for both ion species (Figure 5).

**Figure 6:**
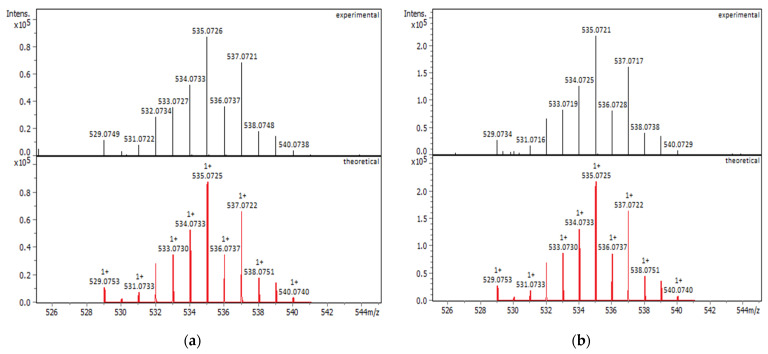
(**a**) ESI-QTOF-MS spectrum of **3-Cl** in methanol (theoretical spectrum in red line); (**b**) ESI-QTOF-MS spectrum of **3-PF_6_** in methanol (theoretical spectrum in red line).

Finally, for **3-Cl** and **3-PF_6_** complexes, the dominant peaks at m/z 535.0726 and *m*/*z* 535.0721 that were detected are those assigned to [C_26_H_26_ClN_2_O_2_Ru]^+^ with mass error at 0.1 mDa and at −0.4 mDa, respectively, and good isotopic pattern fitting (Figure 6).

### 2.2. Evaluation of Biological Activity

**Figure 7:**
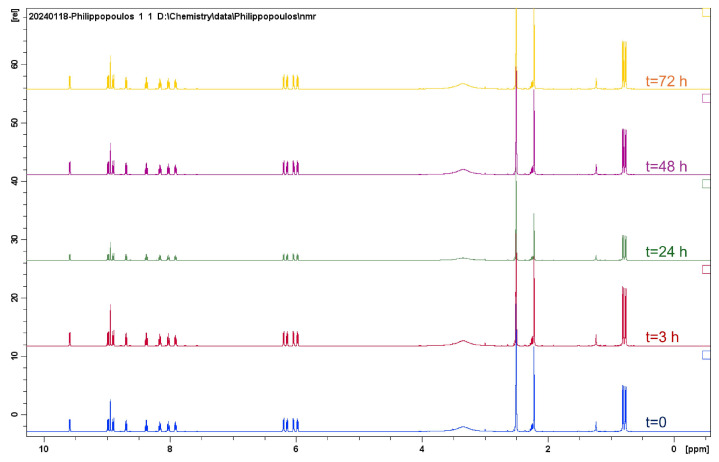
Stability of **2-Cl** in DMSO-d_6_, over time as checked by ^1^H NMR spectroscopy.

The cytotoxicity studies of the organometallic ruthenium(II) complexes **1-Cl, 2-Cl,** and **3-PF_6_** were carried out in dimethyl sulfoxide (DMSO) while **3-Cl** was dissolved in water. According to UV–Vis spectroscopy, complexes **1-Cl** and **2-Cl** are stable in the media that are used for the biological assays (Figures S25–S26), while **3-Cl** undergoes hydrolysis approximately 3 h after with isosbestic points at 276 nm and 322 nm, respectively (Figure S27). The stability of **1-Cl** (Figure S28) and **2-Cl** was also checked by means of ^1^H NMR spectroscopy in DMSO-d_6_, revealing that both complexes are stable in this medium. Notably, for **2-Cl**, ^1^H NMR monitoring was followed over a period of several hours (more than 72 h), showing its remarkable stability in this medium (Figure 7). In any case, for the biological assays, freshly prepared solutions were prepared that were utilized within approximately 5–10 min.

#### Cell Viability Assay 

Cell viability tests of the ligand and metal precursors (pqhyme, pqca, pcqame, [Ru(η^6^-*p*-cymene)(μ-Cl)Cl]_2_) and the ruthenium(II)–arene complexes **1-Cl, 2-Cl** and **3-Cl** were conducted in two cell lines, including epithelial-like HEK293T cells (human embryonic kidney cells) and tumor-derived HeLa cells (cervical cancer cells), by MTT colorimetric assay, after 48 h incubation (MTT = 3-(4,5-dimethylthiazol-2-yl)-2,5-diphenyl tetrazolium bromide). For comparison, the **3-PF_6_** analog was also studied. Generally, a 5 to 10 mM stock solution of the complexes in DMSO (H_2_O for cisplatin) was prepared and diluted appropriately to reach the final concentration reported in the Materials and Methods. HeLa cancer cells were chosen since this cell line is widely used in cytotoxicity studies [54,55] while the non-cancerous HEK293T cell line was used for comparison. Cytotoxicity results of all substances studied in both cell lines expressed as IC_50_ mean values, are summarized in Table 1, including that of cisplatin, which served as control under the same conditions. The IC_50_ values were calculated using a dose–response model, which was obtained from sigmoidal fitting of dose–response curves [56].

**Figure 8:**
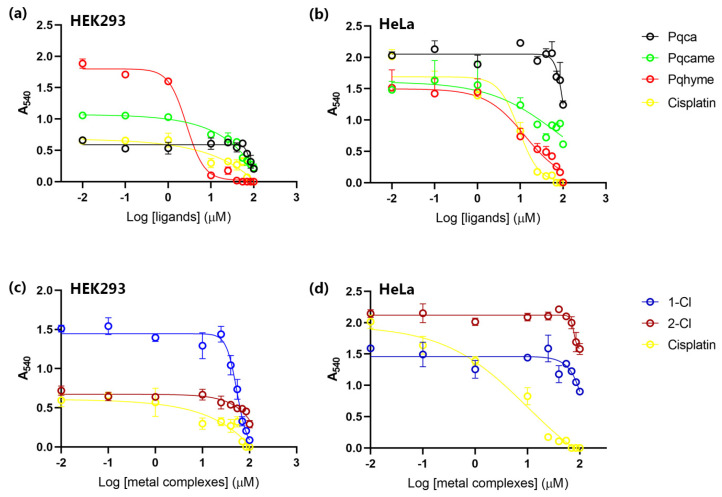
Viability curves comparing the ligands (**a**,**b**) or the complexes (**c**,**d**) with cisplatin in HEK293T (**a**,**c**) and HeLa (**b**,**d**) cell lines.

**Figure 9:**
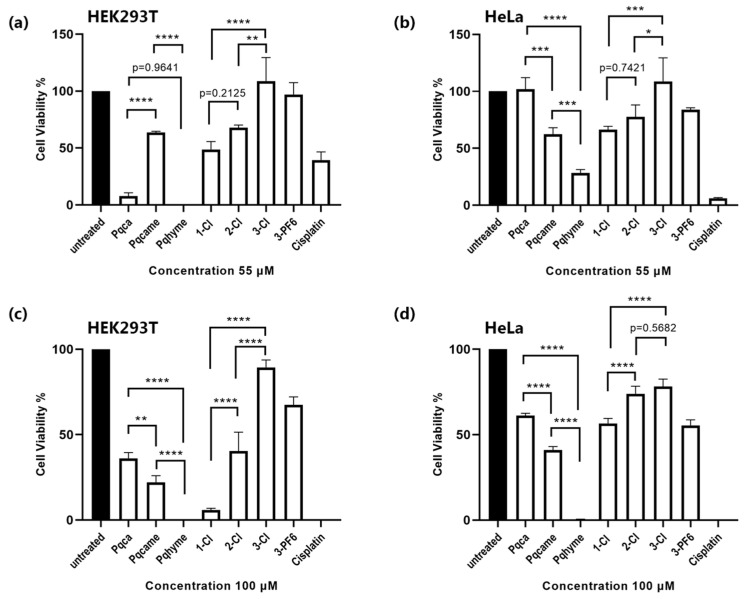
Cell viability effects in HEK293T (**a**,**c**) and HeLa (**b**,**d**) cells following treatment with the compounds with an intermediate (55 μM, (**a**,**b**)) or the maximum (100 μM, (**c**,**d**)) concentration used in the study. Statistics were performed comparing all ligands (Pqca, Pqcame, Pqhyme) and all Ru complexes (1-Cl, 2-Cl, 3-Cl) using one-way ANOVA followed by Tukey’s multiple comparison test. Significant comparisons were marked with asterisks (**** *p* < 0.0001, *** *p* < 0.0008, * *p* = 0.0424, ** *p* = 0.0022 and 0.094 for 2-Cl vs. 3-Cl in (**a**) and for Pqca vs. Pqcame in (**c**), respectively). *p* values for non-significant comparisons are noted in each graph.

From the results listed in Table 1 and Figure 8 and Figure 9, it can be clearly seen that on the HEK293T cell line, the ruthenium(II) analogs bearing the -COOH and -COOMe groups, namely **2-Cl**, **3-Cl,** and **3-PF_6_**, are less potent. Within the series of the complexes tested, cytotoxicity is significantly lower than that of cisplatin, with **1-Cl** being the most potent, followed by **2-Cl**. Notably, the pqhyme ligand incorporating the -CH_2_OH moiety is the most cytotoxic compound, with an IC_50_ value of 2.67 ± 1.23 μM, exhibiting higher cytotoxicity than that of the control (cisplatin, IC_50_ = 16.41 ± 7.62 μM). This potency can also be observed by comparing cell viability after cell treatment with 55 μM of pqhyme and cisplatin, respectively (Figure 9a). Accordingly, cytotoxicity in this cell line decreases in the following order: pqhyme > cisplatin **>** pqcame ≅ **[Ru(*p*-cymene)Cl_2_]_2_ >** pqca **> 3-Cl > 1-Cl** > **3-PF_6_** > **2-Cl**. 

In tumor-derived HeLa cells, the ligand precursors pqhyme, pqca, and pqcame showed variable cytotoxic activities, with that of pqhyme being approximately ten-fold lower compared to cisplatin. In contrast, the ruthenium(II) complexes were less potent, with a cytotoxicity profile that drops in the following order: cisplatin > pqhyme > pqcame > **[Ru(p-cymene)Cl_2_]_2_** > pqca > **3-Cl > 1-Cl** > **3-PF_6_** > **2-Cl**. 

A comparison of the maximum concentration used (100 μM) with an intermediate concentration (55 μM) indicated a dose-dependent reduction in cell viability in all compounds tested in these cells (Figure 9b,d). Moreover, in this cell line, the complexes reported herein were more effective compared to [Ru(*p*-cymene)Cl_2_(μ-(4ampy)], 4ampy = 4-aminopyridine, which display an almost 16-fold lower IC_50_ value (1.6 ± 0.0 mM) [57]. They also present similar potencies to those reported for a series of neutral and cationic complexes of the type [(η^6^-*p*-cymene)RuCl_2_L], L = 2-aminophenol (IC_50_ = 82.9 μM), 4-aminophenol (IC_50_ = 171.1 μM), and [(*η*^6^-*p*-cymene)RuClL_2_]PF_6_; L = 72-aminophenol(IC_50_ = 57.6 μM) [56]. The observed results on this cancer cell line could be cautiously compared with those reported for other similar ruthenium–arene (indan as the arene) complexes containing 2,2′-bipyridine (IC_50_ > 100 μM, against A2780 cells) [58].

The results further suggested that in both cell lines, the cytotoxicity of the free organic ligands was significantly higher than that of the relevant ruthenium(II) complexes. In addition, all complexes showed higher cytotoxicity against the healthy cell line, which at first glance, is not in favor of their possible use as anticancer agents. However, recent reports highlight that cytotoxicity differs within a selected panel of different cell lines examined [56]. In fact, the very similar complex [Ru(N^N)(*p*-cymene)Cl]Cl where N^N stands for the 4,4′-dicarboxy-2,2′-bipyridine ligand, exhibits high selectivity toward carcinoma human T24 bladder cells and is not toxic to other cell lines [59]. 

## 3. Materials and Methods

The synthesis of the ruthenium(II) complexes was carried out under an argon atmosphere using standard Schlenk techniques while all organic ligands were prepared under aerobic conditions. All solvents were of analytical grade, distilled, and dried before use according to standard methods. RuCl_3_∙H_2_O and α-terpinene were purchased from Riedel de Haën and Sigma-Aldrich, respectively. Ligand precursors 4-hydroxymethyl-2-(pyridin-2-yl)quinoline (pqhyme) [38], 4-carboxy-2-(pyridin-2-yl)quinoline (pqca) [60,61] and 4-carboxymethyl-2-(pyridin-2-yl)quinoline (pqcame) were prepared according to the published procedures [62]. The ruthenium(II) dinuclear complex [Ru(η^6^-*p*-cymene)(μ-Cl)Cl]_2_ was synthesized according to the literature reports [48]. The infrared spectra were recorded on IR Affinity-1 SHIMADZU in the spectral range 4000–400 cm^−1^, in the ATR mode. Elemental analyses were obtained from Microanalysis Center of Institut für Anorganische Chemie Universität Bonn. ^1^H and ^13^C NMR spectra were recorded on Bruker Avance NEO 500 and 400 MHz systems (magnet ASCEND 11.76 Tesla) operating at 500.11 MHz (400 MHz) for ^1^H, 125.75 (100 MHz) for ^13^C, and controlled by TopSpin 4.1.4. Experiments were acquired at 300 K unless stated otherwise, and temperature was controlled by Bruker BCU II Cooler unit. Samples were prepared and loaded in 5 mm NMR tubes and measured with a double resonance broadband inverse (BBI) detection probe or broadband observe (BBO H&F) Prodigy cryoprobe (Bruker, Germany). NMR spectra of metal complexes were assigned using the ^1^H-^1^H COSY, ^1^H-^13^C HSQC, and ^1^H-^13^C HMBC methods. One and two-dimensional spectra were acquired with Bruker’s standard pulse programs while proton spectra with pressaturation of the residual water signal were acquired with optimization of the ^1^H 90° pulse length and processed with an exponential window function and a line broadening factor 0.3 prior to Fourier transformation. *J* values are given in Hz. Absorption spectra were recorded with a CARY 3E UV–Vis spectrometer. X-ray diffractions were collected on a dual source (IμS Diamond Cu/Κα and Mo/Κα) Bruker D8-Venture SC-XRD instrument equipped with a Photon-III area detector at 100K using an Oxford Cryosystems 100 cryostream. Melting or decomposition points were determined with a Büchi SMP 530 (Büchi Labortechnik AG, Flawil, Switzerland) melting point apparatus and were not corrected. The samples were sealed in capillary tubes and heated slowly until the compounds melted or decomposed. Molar conductance measurements were performed twice in a WTW-LF3 conductive meter. All data were corrected with the specific conductivity of the solvent. The correction was made by subtracting the specific conductivity of the solvent medium from those of the solutions. The molar conductivities (*Λ*/S cm^2^ mol^−1^) were calculated from the experimental specific conductivities (*k*/S cm^1^) and the concentrations (c/mol L) of the solutions using the equation *Λ* = 10^3^ *k*/c. The synthesized ruthenium complexes were diluted in methanol to prepare solutions at concentration of 1 mgL^−1^. The analysis of the solutions was performed using an ESI-QTOF-MS system (Maxis Impact, Bruker Daltonics, Bremen, Germany). The ESI source operated in positive mode with the following parameters: capillary voltage 4500; end plate offset 500 V; nebulizer pressure 0.4 bar; drying gas 4 L min^−1^; gas temperature 180 °C. The QTOF-MS operated in full-scan mode and recorded spectra over the range of *m*/*z* 50–1000. External mass calibration was performed before analysis using a mixture of sodium formate clusters. The instrument provided a typical resolving power between 36,000 and 40,000 at *m*/*z* 226.1593, 430.9137, and 702.8636. During data treatment, using Bruker’s software (DataAnalysis 5.3 and IsotopePattern tool) internal mass calibration was first performed, and then background signal subtraction, so that a high-quality mass spectrum was obtained. For identifying a compound, the following criteria were evaluated: |mass accuracy| < 5 mDa; isotopic pattern fitting < 50 mSigma (the lower the value, the better the fitting of theoretical and experimental isotopic patterns.)

### 3.1. Synthesis and Characterization

#### 3.1.1. Synthesis of complexes **1-Cl**, **2-Cl**, **3-Cl**

In a Schleck tube and under an argon atmosphere [Ru(η^6^-*p*-cymene)(μ-Cl)Cl]_2_ (1 mol equiv.) and 2.1 mol equivalents of the appropriate bidentate ligand (L) were added. Dry methanol (10 mL) was added, and the mixture was stirred for approximately 18 to 20 h at ambient temperature, to ensure completion of the reaction. After filtration (in the air), the volume of the solution was reduced to approximately 2–3 milliliters, and diethyl ether (10 mL) was added. The solid precipitated was treated with diethyl ether (2 × 5 mL) and dried in vacuo at 60 °C.

#### 3.1.2. Data for Complexes **1-Cl**, **2-Cl**, and **3-Cl**

[Ru(η^6^-*p*-cymene)(pqhyme)Cl][Cl] **(1-Cl)**: Yield: 61 mg (85%). m.p. 150 °C. 155 °C (dec). Anal. Calcd for C_25_H_26_Cl_2_N_2_ORu∙(H_2_O): C, 53.57; H, 5.04; N, 5.00. Found: C, 53.62; H, 5.14; N, 5.20%. FT-IR (ATR mode): ν[cm^−1^] = 3345 (br) [ν(O-H)], [3061(w) [ν(C-H)_arom_)], 2996(m) [ν(C-H)_aliph_], 2998-2850(m) [ν(C-H)_aliph_], 1551(s) [ν(C=C)], 804(m) [ν(Ru-C)], 796(s), 771(vs) [δ(C-H) out of plane], 744(s), 538(m). UV–Vis (DMSO, *λ*_max_, nm, 10^−4^ M): 439 (*ε* = 900 M^−1^ cm^−1^), 354 (*ε* = 2970 M^−1^ cm^−1^), 296 (*ε* = 4970 M^−1^ cm^−1^). *Λ*(DMSO): 21 S cm^2^ mol^−1^. ESI-QTOF-MS (MeOH, positive mode): *m/z* 507.0774 detected for [C_25_H_26_ON_2_ClRu]^+^ (calc. 507.0775, mass error −0.1mDa). ^1^H NMR (CDCl_3_, 500 MHz, 298 K): *δ* 0.87 (d, *J* = 8.0, 3H, CH(C*H*_3_)_2,_), 0.91 (d, *J* = 8.0, 3H, CH(C*H*_3_)_2_), 2.25 (s, 3H, CH_3_-cym), 2.29 (sept, *J* = 4.0, 1H, C*H*(CH_3_)_2_)_,_ 5.47 (s, 2H, C*H_2_*-OH), 5.61 (d, *J* = 7.0, 1H, H-cym_ar_), 5.71 (d, *J* = 7.0, 1H, H-cym_ar_), 5.86 (d, J = 7.0, 1H, H-cym_ar_), 5.92 (d, *J* = 7.0, 1H, H-cym_ar_), 7.63 (t, *J* = 8.0, 1H, H7), 7.75 (t, *J* = 8.0, 1H, H5′), 7.92 (m, 2H, H4′, H6), 8.05 (d, *J* = 8.0, 1H, H3′), 8.58 (d, *J* = 8.0, 1H, H5), 8.77 (d, *J* = 8.0, 1H, H6′), 8.94 (s, 1H, H3), 9.58 (d, *J* = 8.0, 1H, H8). ^1^H NMR (DMSO-d_6_, 400 MHz, 298 K): δ 0.50 (d, *J* = 7, 6H, CH(CH_3_)_2_), 2.20 (s, 3H, CH_3_-cym), 2.25 (m, 1H, CH(CH_3_)_2_), 5.30 (br.s, 2H, CH_2_-OH), 6.02 (d, *J* = 7.0, 2H, H-cym_ar_), 6.10 (d, *J* = 7.0, 2H, H-cym_ar_), 7.89 (t, *J* = 8.0, 1H); 7.99 (t, *J* = 8.0, 1H); 8.19 (t, *J* = 8.0, 1H); 8.30 (t, *J* = 8.0, 1H), 8.48 (t, *J* = 8.0, 1H), 8.60 (s, 1H), 8.80 (m, 2H), 9.60 (s, 1H). ^13^C{^1^H} NMR (125.75 MHz, CDCl_3_) *δ*_C_/ppm 18.68 (CH-(*C*H_3_)_2_), 21.76 (CH-(*C*H_3_)_2_), 22.32 (CH-(*C*H_3_)_2_), 31.01 (*C*H-(CH_3_)_2_), 60.38 (CH_2_OH), 85.15 (C-C_cym-ar_), 85.34 (C-C_cym-ar_), 85.52 (C-C_cym-ar_), 86.74 (C-C_cym-ar_), 103.60 (*C*_cym_-CH-(CH_3_)_2_), 105.87 (CH_3_-Ccym), 117.67 (C3), 124.55 (C3′),125.29 (C4), 127.29 (C7), 128.08 (C5), 129.20 (C5′), 129.63 (C6), 131.68 (C4a), 140.10 (C2), 148.50 (C4′), 155.80 (C2′), 155.85 (C8a), 155.99 (C8, C6′).

[Ru(η^6^-*p*-cymene)(pqca)Cl][Cl] **(2-Cl)**: Yield: 122 mg (85%). m.p. 125 °C. 150 (dec). (Anal. Calcd for C_25_H_24_Cl_2_N_2_O_2_Ru∙2(H_2_O): C, 50.68; H, 4.76; N, 4.73. Found: C, 50.81; H, 4.35; N, 4.74%. FT-IR (ATR mode): ν[cm^−1^] *=* 3363 (br) [ν(O-H) lattice water and COOH ], 3055(w) [ν(C-H)_arom_)], 2971(m) [ν(C-H)_aliph_], 1704(s) [ν_as_(C=O)], 1597(m) [ν(C=C)], 1545(m) [ν(C=C)], 1474(m) [ν(C=N)], 1200(s) [ν_s_(C-O)], 1054(w) [δ(C-N)], 793 (m) [ν(Ru-C)], 771(s) [δ(C-H) out of plane]. UV–Vis (DMSO, *λ*_max_, nm, 10^−4^ M): 435 (*ε* = 1500 M^−1^ cm^−1^), 357 (*ε* = 5400 M^−1^ cm^−1^), 298 (*ε* = 8700 M^−1^ cm^−1^). *Λ*(DMSO): 54 S cm^2^ mol^−1^. ESI-QTOF-MS (MeOH, positive mode): *m/z* 521.0579 detected for [C_25_H_24_N_2_ClO_2_Ru]^+^ (calc. 521.0568 mass error, 1.1 mDa), *m/z* 543.0399 detected for [C_25_H_23_N_2_NaClO_2_Ru]^+^ (calc. 543.0399 543.0387, mass error, 1.2 mDa). ^1^H NMR (DMSO-d_6_, 500 MHz, 298 K): *δ* 0.77 (d, *J* = 7.0, 3H, CH(C*H*_3_)_2_), 0.81 (d, *J* = 7.0, 3H, CH(C*H*_3_)_2_), 2.22 (s, 1H, CH_3_-cym), 2.25 (sept, 3H, C*H*(CH_3_)_2_ overlapped with CH_3_-cym), 5.97 (d, *J* = 7.0, 1H, H-cym_ar_ ), 6.04 (d, *J* = 7.0, 1H, H-cym_ar_), 6.14 (d, *J* = 7.0, 1H, H-cym_ar_), 6.20 (d, *J* = 7.0, 1H, H-cym_ar_), 7.91 (t, *J* = 8.0, 1H, H6′), 8.03 (t, *J* = 8.0, 1H, H4′), 8.16 (t, *J* = 8.0, 1H, H5′), 8.37 (t, *J* = 8.0, 1H, H6), 8.70 (t, *J* = 8.0, 1H H3′), 8.90 (d, *J* = 8.0, 1H, H6′), 8.94 (s, 1H, H3), 8.98 (d, *J* = 8.0, 1H, H6), 9.60 (d, *J* = 8.0, 1H, H8). ^13^C{^1^H} NMR (125.75 MHz, Me_2_CO-d_6_) *δ*_C_/ppm 18.18 (*C*H_3_-cym), 21.21 (CH-(*C*H_3_)_2_), 21.58 (CH-(*C*H_3_)_2_), 30.26 (*C*H-(CH_3_)_2_), 84.15 (C-C_cym-ar_), 85.24 (C-C_cym-ar_), 86.08 (C-C_cym-ar_), 87.44 (C-C_cym-ar_), 104.43 (*C*_cym_-CH-(CH_3_)_2_), 104.81 (CH_3_-*C*_cym_), 118.72 (C3), 125.29 (C4′), 125.78 (C3′), 126.33 (C7), 128.37 (C6), 130.46 (C5), 132.64 (C5′), 140.16 (C5), 142.09 (C4), 149.33 (C2′), 154.31 (C8a), 155.84 (C2), 156.36 (C8), 162.92 (8a), 166.31 (CO).

[Ru(η^6^-*p*-cymene)(pqcame)Cl][Cl] **(3-Cl)**: Yield: 80 mg (92%). m.p. 111 °C (dec). Anal. Calcd for C_26_H_26_Cl_2_N_2_ORu∙2(H_2_O): C, 52.88; H, 5.12; N, 4.74. Found: C, 52.87; H, 5.39; N, 4.85%. FT-IR (ATR mode): ν[cm^−1^] = 3374 (br) [ν(O-H)], 3056 (w) [ν(C-H)_arom_)], 2966-2872 (m) [ν(C-H)_aliph_], 1723(s) [ν_as_(C=O)], 1597(m) [ν(C=C)], 1479(m) [ν(C=N)], 1370(m) [δ(C-H)], 1263(m), 1211(m) [ν_s_(C-O)], 1146(m) [ν(O=)C–CH_3_], 799(w) [ν(Ru-C)], 773(s) [δ(C-H) out of plane], 746(m). UV–Vis (H_2_O, *λ*_max_, nm, 7.8 ×10^−5^ M): 422 (*ε* = 1500 M^−1^ cm^−1^), 357 (*ε* = 6400 M^−1^ cm^−1^), 293 (*ε* = 9700 M^−1^ cm^−1^), 258 (*ε* = 9600 M^−1^ cm^−1^). *Λ*(H_2_O): 100 S cm^2^ mol^−1^. ESI-QTOF-MS (MeOH, positive mode): *m/z* 535.0726 detected for [C_26_H_26_O_2_N_2_ClRu]^+^ (calc. 535.0725, mass error, 0.1 mDa). ^1^H NMR (CDCl_3_, 500 MHz, 298 K): *δ* 0.88 (d, *J* = 5.0, 3H, CH(C*H*_3_)_2,_), 0.93 (d, *J* = 5.0, 3H, CH(C*H*_3_)_2_), 2.23 (s, 3H, CH_3_-cym), 2.36 (sept, *J* = 5.0, 1H, C*H*(CH_3_)_2_)_,_ 4.15 (s, 3H, COOC*H*_3_), 5.78 (d, *J* = 5.0, 1H, H-cym_ar_), 6.01 (d, *J* = 5.0, 1H, H-cym_ar_), 6.27 (d, *J* = 5.0, 1H, H-cym_ar_), 6.35 (d, *J* = 5.0, 1H, H-cym_ar_), 7.91 (m, 2H, H7, H5′), 8.05 (t, *J* = 10.0, 1H, H4′), 8.16 (t, *J* = 10.0, 1H, H6), 8.46 (d, *J* = 10.0, 1H, H5); 8.74 (s, 1H, H3 ); 8.93 (d, *J* = 10.0, 1H, H3′); 8.97 (d, *J* = 10.0, 1H, H6); 9.95 (s, 1H, H8). ^13^C{^1^H} NMR (125.75 MHz, CDCl_3_) *δ*_C_/ppm 18.98 (*C*H_3_-cym), 21.82 (CH-(*C*H_3_)_2_), 22.45 (CH-(*C*H_3_)_2_), 31.25 (*C*H-(CH_3_)_2_), 53.90(COO*C*H_3_ ), 86.01 (s, C-C_cym-ar_), 86.32 (C-C_cym-ar_), 86.38 (s, C-C_cym-ar_), 88.36 (s, C-C_cym-ar_), 104.73 (*C*_cym_-CH-(CH_3_)_2_), 106.20 (CH_3_-*C*_cym_), 119.78 (C3 ), 124.92 (C8), 126.32 (C3′), 126.99 (C7), 130.02 (C4′), 130.34 (C5′),131.11 (C6′), 132.73 (C6), 138.36 (C8a), 139.95 (C4a), 150.67 (C2′),153.71 (C2), 156.29 (C4), 158.88 (C6), 164.61 (CO).

#### 3.1.3. Synthesis of Complexes **1-PF_6_**, **3-PF_6_**

Under aerobic conditions, the obtained chlorido complex (**1-Cl** or **3-Cl**) was dissolved in water (5 mL) and a few drops of a saturated aqueous solution of KPF_6 (aq)_ were added. Upon addition, a yellow precipitate appeared, and the mixture was stirred for 30 min further, to ensure completion of the reaction. The yellow solid was collected via vacuum filtration, was washed subsequently with water (5 mL) and diethyl ether (2 × 5 mL), and dried in vacuo at 60 °C. 

#### 3.1.4. Data for complexes **1-PF_6_**, **3-PF_6_**

[Ru(η^6^-*p*-cymene)(pqhyme)Cl][PF_6_] (**1-PF_6_**): Yield: 51 mg (85%). m.p. 130 °C (dec). Anal. Calcd for C_25_H_26_ClF_6_N_2_OPRu: C, 46.05; H, 4.02; N, 4.30. Found: C, 46.27; H, 4.09; N, 4.25%. FT-IR (ATR mode): ν[cm^−1^] = 3096(w) [ν(C-H)_arom_)], 2970-2876(m) [ν(C-H)_aliph_], 1601(s) [ν(C=C)], 1486(m) [ν(C=C)], 1371(w) [ν(C=C)], 832 (vs) [ν(P-F)], 792(s), 745(s) [δ (C-H) out of plane], 557(s) [ν(P-F)]). UV–Vis (Me_2_CO, λ_max_, nm, 8.6 × 10^−5^ M): 422 (ε = 2000 M^−1^ cm^−1^), 372 (ε = 2700 M^−1^ cm^−1^), 349 (ε = 9400 M^−1^ cm^−1^), 335 (ε = 8800 M^−1^ cm^−1^). *Λ*(DMSO): 22 S cm^2^ mol^−1^. ESI-QTOF-MS (MeOH, positive mode): *m/z* 507.0770 detected for [C_25_H_26_ON_2_ClRu]^+^ (calc. 507.0775, mass error, −0.5 mDa). ^1^H NMR (Me_2_CO-d_6_, 500 MHz, 298 K): *δ* 0.93 (d, *J* = 5.0, 3H, CH(C*H*_3_)_2_), 0.97 (d, *J* = 5.0, 3H, CH(C*H*_3_)_2_), 2.30 (s, 3H, CH_3_-cym), 2.44 (sept, *J* = 5.0, 1H, C*H*(CH_3_)_2_), 5.48 (d, *J* = 5.0, 2H, C*H_2_*-OH), 6.01 (m, 2H, H-cym_ar_), 6.10 (d, *J* = 5.0, 1H, H-cym_ar_), 6.19 (d, *J* = 5.0, 1H, H-cym_ar_), 7.91 (t, *J* = 10.0, 1H, H7), 7.94 (t, *J* = 10.0, 1H, H4′); 8.10 (t, *J* = 5.0, 1H, H5′), 8.29 (d, *J* = 10.0, 1H, H5), 8.42 (t, *J* = 5.0, 1H, H6), 8.73 (s, 1H, H3), 8.85 (d, *J* = 5.0, 1H, H3′), 9.02 (d, *J* = 5.0, 1H, H6′), 9.63 (d, *J* = 5.0, 1H, H8). ^13^C{^1^H} NMR (125.75 MHz, Me_2_CO-d_6_) *δ*_C_/ppm 18.73 (*C*H_3_-cym), 21.87 (CH-(*C*H_3_)_2_), 22.32 (CH-(*C*H_3_)_2_), 31.76 (*C*H-(CH_3_)_2_), 61.08 (CH_2_OH), 85.79 (C-C_cym-ar_), 86.43 (C-C_cym-ar_), 86.84 (C-C_cym-ar_), 87.85 (C-C_cym-ar_), 105.28 (CH_3_-*C*_cym_), 106.50 (CH_3_-*C*_cym_), 116.55 (*C*_cym_-CH-(CH_3_)_2_), 125.03(C3), 126.04 (C3′),127.85 (C4), 129.02 (C7), 130.37 (C5), 131.68 (C5′), 132.98 (C6), 141.09 (C4α), 149.61 (C2), 154.75 (C4′), 156.49 (C2′), 156.98 (C8a), 157.17 (C8, C6′).

[Ru(η^6^-*p*-cymene)(pqcame)Cl][PF_6_] (**3-PF_6_**): Yield: (0.079 g, 75%). m.p. 190 °C (dec). Found: C, 45.25; H, 4.00; N, 3.97. C_26_H_26_ClF_6_N_2_O_2_PRu∙0.6H_2_O requires C, 45.21; H, 3.97; N 4.06%. FT-IR (ATR mode): ν[cm^−1^] = 3067(w) [ν(C–H)_arom_], 2873(w) [ν_as_(C–H)_aliph_], 2852(w) [ν_s_(C–H)_aliph_], 1727(vs) [ν_as_(C=O)], 1594(w) [ν(C=N)], 1484(w) [ν(C=C)], 1371(w) [ν(C=C)], 1218(m) [ν_s_(C-O)], 825 (vs) [ν(P–F)], 798(w) [ν(Ru–C) probably overlapped with ν(P–F)), 555 (s) [ν(P–F)]. UV–Vis (Me_2_CO, *λ*_max_, nm, 1.0 × 10^−4^): 436 (*ε* = 3100 M^−1^ cm^−1^), 354 (*ε* = 9700 M^−1^ cm^−1^). *Λ*(Me_2_CO): 103 S cm^2^ mol^−1^. ESI-QTOF-MS (MeOH, positive mode): *m/z* 535.0721 detected for [C_26_H_26_N_2_ClO_2_Ru]^+^ (calc. 535.0725, mass error, −0.4 mDa). ^1^H NMR (500 MHz, Me_2_CO-d_6_): *δ* 0.94 (d, *J* = 8.0, 3H, CH(C*H*_3_)_2_), 0.98 (d, *J* = 8.0, 3H, CH(C*H*_3_)_2_), 2.30 (s, 3H, C*H*_3_-cym), 2.49 (sept, *J* = 8.0, 1H, C*H*(*C*H_3_)_2_ ), 4.14 (s, 3H, COOCH_3_), 6.04 (m, 2H, H-cym_ar_), 6.04 (m, 2H, H-cym_ar_), 6.13 (d, *J* = 4.0, 1H, H-cym_ar_), 6.23 (d, *J* = 4.0, 1H, H-cym_ar_), 7.94 (t, *J* = 4.0, 1H, H7), 8.04 (t, *J* = 4.0, 1H, H5′), 8.17 (t, *J* = 4.0, 1H, H4′), 8.42 (t, *J* = 8.0, 1H, H6), 8.85 (d, *J* = 8.0, 1H, H5), 8.91 (d, *J* = 8.0, 1H, H8), 9.02 (s, 1H, H3), 9.13 (d, *J* = 4.0, 1H, H3′), 9.63 (d, *J* = 4.0, 1H, H6′). ^13^C{^1^H} NMR (125.75 MHz, Me_2_CO-d_6_): *δ* 18.87 (*C*H_3_-cym), 22.11 (CH-(*C*H_3_)_2_), 22.50 (CH-(*C*H_3_)_2_), 31.98 (*C*H-(CH_3_)_2_), 54.13 (COO*C*H_3_), 86.38 (C-C_cym-ar_), 86.66 (C-C_cym-ar_), 87.05 (C-C_cym-ar_), 88.36 (C-C_cym-ar_), 105.74 (CH_3_-*C*_cym_), 107.56 (*C*_cym_-CH-(CH_3_)_2_), 120.91 (C3), 126.76 (C8), 127.11 (C4a), 127.52 (C5), 131.91 (C3′),132.04 (C5′), 133.81 (C4′), 140.47 (C7), 141.35 (C6), 150,17 (C4), 151.45 (C8a), 155.95 (C2), 157.24 (C6′), 157.47 (C2′), 166.00 (CO).

### 3.2. Single-Crystal X-ray Structural Determination

Data for both **1-PF_6_** and **3-PF_6_** were collected on a dual source (IμS Diamond Cu/Κα and Mo/Κα) Bruker D8-Venture SC-XRD instrument equipped with a Photon-III area detector at 100K using an Oxford Cryosystems 100 cryostream. Data were collected using φ and ω scans to fill the Ewald sphere using a 4-circle kappa goniometer. Data collection and subsequent processing were handled by the APEX4 software package (2021.10 (version 10)). A multi-scan absorption correction (SADABS 2016/2) was applied in both cases. Data solution [63] and model refinement [64,65] were achieved using the Olex2-1.5 software package [66]. All atoms were refined anisotropically and hydrogen atoms were added using the riding model. For **1-PF_6_**, data were collected using Mo/Κα to a resolution of 0.75 Å. The PF_6_^−^ counter anion shows significant occupational disorder over two sites of its fluorine atoms. This disorder was modeled using the PART command and the site occupation was refined freely to ca 54:46. Fluorine atoms F5 and F13 were refined isotropically using the ISOR command. Furthermore, the ADP of atom C5 of the *p*-cymene ligand was restrained to the same value of atom C2 of the same ligand using the EADP command, since attempts to refine the former isotropically led to a non-converging refinement. The O-H hydrogen atom was found in the Fourier difference map and its position was refined freely. In the case of **3-PF_6_** data were collected using Cu/Κα to a resolution of 0.81 Å. Original unit cell refinement pointed to the non-standard setting monoclinic P2_1_/n space group; although data solution and final model refinement were achievable, Goof was quite high (>1.3) while the number of bad equivalents and systematic absences were exceptionally high. Upon careful reprocessing of the data, it was found that the true symmetry of the unit cell was triclinic (SG *P-1*) with γ almost 90° and with two molecules in the asymmetric unit. Reprocessing of the data in *P-1* resulted in better collection statistics (Rint 0.0603 vs. 0.0713 in *P2_1_/n*) while final model refinement was not problematic. Both PF_6_^−^ counter anions show occupational disorder which was modeled using the PART command and the occupancies refined freely to ca 79:21 and 90:10. Some fluorine atoms’ ADPs had to be restrained to the ADPs of their counterparts in the corresponding PART using the EADP command. The model was refined as a two-component (72:28 twin fraction) pseudo-merohedral twin (TWIN LAW: 1 0 0 0 −1 0 0 0 −1 as derived from PLATON TWINROTMAT). Finally, residual electron density of 1.6 e.Å^−3^ was found 0.75 Å from atom Ru2 and has no chemical meaning and is attributed to absorption artifacts (analytical absorption correction based on crystal faces did not mitigate the situation). A summary of the crystal data, data collection, and refinement for the structures of complexes **1-PF_6_** and **3-PF_6_** is given in Table 2. CCDC 2347571 (**1-PF6**) and 2347570 (**3-PF_6_**), respectively, contain the supplementary crystallographic data for this paper. These data can be obtained free of charge from the Cambridge Crystallographic Data Centre via www.ccdc.cam.ac.uk/data_reqeust/cif (accessed on 11 April 2024). 

### 3.3. Biological Evaluation

#### 3.3.1. Cell Lines

The HEK293T cell line (immortalized human embryonic kidney cells) and the HeLa cell line (cervical cancer cells) were obtained from ATCC. Cells were cultured at 37 °C and 5% CO_2_ in high glucose Dulbecco’s modified Eagle’s medium (DMEM) containing 10% fetal bovine serum (FBS), penicillin (100 units/mL), and streptomycin (100 μg/mL).

#### 3.3.2. MTT Assay

Ruthenium complexes were diluted in stock solutions of 10 mM in dimethyl sulfoxide (DMSO). A similar concentration of cisplatin dissolved in H_2_O was used. Cells were seeded in sterile tissue culture 96-well plates at a density of 3,000 cells per well. After 24 h, the medium was removed and replaced with fresh media containing various concentrations (0.01 μΜ, 0.1 μΜ, 1 μΜ, 10 μΜ, 25 μΜ, 40 μΜ, 55 μΜ, 70 μΜ, 85 μΜ, and 100 μΜ) of the compounds to be tested and were further incubated for 48 h at 37 °C. DMSO was used as vehicle at ≤1%. Culture medium containing the compounds (or DMSO) was removed and 100 μL of 0.5 mg/mL MTT reagent (Applichem) diluted in DMEM was added to the cells for 3 h at 37 °C. After 3 h incubation, MTT was removed and 200 μL of DMSO was added. Absorbance values of formazan were measured at 540 nm using a BioTek Synergy H1 microplate reader (Agilent). All compounds were tested in triplicate. The results were statistically analyzed using the nonlinear regression by GraphPad PRISM (Version 9) to extract IC_50_ values.

## 4. Conclusions

In summary, in this study, we report, for the first time, on the coordination chemistry and cytotoxicity properties of a series of half-sandwich organometallic ruthenium(II) complexes incorporating substituted pyridine–quinoline ligands (N^N) with -CH_2_OH, -CO_2_H, and -CO_2_Me pending groups, in the 4-position of quinoline. The cytotoxic activities of the ruthenium(II)–arene chloride analogs, in tumor-derived HeLa cells are poor, while cytotoxicity of the organic ligand precursors is significantly higher. This is not a drawback since cytotoxicity differs within the different cell lines examined. The results of the present study not only provided us with the required knowledge about the cytotoxic potencies of ruthenium(II)–arene complexes, bearing substituted pyridine–quinolines but prompted us to continue our initial studies. Further studies with other in vitro cancer cell lines are required along with DNA and/or protein targeting experiments. Research towards this goal is underway, in an effort to design new ruthenium(II) complexes with improved biological profiles. 

## Figures and Tables

**Table 1:** IC_50_ values (μM) of the pyridine–quinoline containing complexes against the HEK293T and Hela cell lines. Cisplatin is used as a control. Values are the mean ± SD.

Compounds	IC_50_ for HEK293T Cells (μM)	IC_50_ for HeLa Cells (μM)
pqhyme	2.67 ± 1.23	12.84 ± 1.01
[Ru(*p*-cymene)Cl_2_]_2_	48.33 ± 1.66	48.90 ± 1.73
**1-Cl**	54.39 ± 1.53	119.40 ± 1.29
pqca	87.48 ± 1.30	100.00 ± 3.47
**2-Cl**	179.60 ± 49.12	162.00 ± 21.30
pqcame	48.85 ± 3.19	56.44 ± 12.68
**3-Cl**	>115	>80
**3-PF_6_**	145.30 ± 16.20	134.40 ± 3.30
cis-platin	16.41 ± 7.62	9.26 ± 1.09

Data (viability curves and cell viability effects) are presented in Figure 8 and Figure 9**.**

**Table 2:** Summary of crystallographic data and structure refinement for complexes **1-PF_6_** and **3-PF_6_**.

Compound	1-PF_6_	3-PF_6_
Color, habit	Yellow, block	Yellow, plate
Size/mm	0.044 × 0.043 × 0.037	0.106 × 0.04 × 0.02
Empirical formula	C_25_H_26_ClF_6_N_2_OPRu	C_26_H_26_ClF_6_N_2_O_2_PRu
FW	651.97	679.98
Crystal system	Monoclinic	Triclinic
Space group	*P2_1_/c*	*P-1*
*a*/Å	11.0604(10)	10.9647(7)
*b*/Å	20.486(2)	11.4231(7)
*c*/Å	10.9695(12)	22.0613(13)
*α*/°	90	102.738(3)
*β*/°	93.802(4)	90.100(3)
*γ*/°	90	90.030(3)
*V*/Å^3^	2480.1(4)	2695.2(3)
*Z*	4	4
μ/mm^−1^	0.873	6.815
*T*/K	100	100
*θ*_min/max/full_ (°)	1.988/28.247/25.242	2.053/72.383/67.679
Completeness to *θ*_max/full_ (%)	99.0/99.6	99.4/100
Reflections total/ Independent	20955/4275	129500/10164
Parameters/restraints	391/12	795/24
*R*_int_	0.1199	0.0603
Final *R*1, *wR*2	0.0477/0.1104	0.0347/0.0881
Goodness-of-fit	0.994	1.074
Largest peak, hole/e.Å^−3^	0.9/-1.2	1.6/−0.9
*ρ_calc_*/g·cm^−3^	1.746	1.676

## Data Availability

The data presented in this study are available on request from the corresponding author.

## Supplementary Materials

The following supporting information can be downloaded at: https://www.mdpi.com/article/10.3390/molecules29133215/s1, Figure S1: ATR spectra of pqhyme and complexes 1-Cl, 1-PF_6_; Figures S2–S7: NMR spectra of complexes 1-Cl, 1-PF_6_; Figure S8: X-ray structure of 1-Cl•2CHCl_3_•pqhyme; Table S1: Crystallographic data of 1-Cl•2CHCl_3_•pqhyme; Figure S9: Hydrogen bonding interactions in the unit cell of 1-Cl•2CHCl_3_•pqhyme; Table S2: A list of hydrogen bonding interactions within the crystal of 1-Cl•2CHCl_3_•pqhyme; Figure S10: Intermolecular non-classical C–H∙∙∙π contacts in 1-PF_6_; Figure S11: ATR spectra of pqca and complexes 2-Cl, 2; Figures S12–S15: NMR spectra of 2-Cl; Figure S16: ATR spectra of pqcame, 3-Cl and 3-PF_6_; Figures S17–S23: NMR spectra of 3-Cl, 3-PF_6_; Figure S24: Intramolecular and intermolecular contacts in 3-PF_6_; Figures S25–S27: UV–Vis spectra of 1-Cl, 2-Cl in DMSO and 3-Cl in H_2_O; Figure S28: Stability of 2-Cl, checked by ^1^H NMR, in DMSO-d_6_ over time.
