# Supplementary material for: Ruthenium p-Cymene Complexes Incorporating Substituted Pyridine–Quinoline-Based Ligands: Synthesis, Characterization, and Cytotoxic Properties
Source: Molecules. 2024 Jul 6;29(13):3215. doi: 10.3390/molecules29133215 (PMC11243419; doi:10.3390/molecules29133215)
Supplement: Supplementary file 1 [file molecules-29-03215-s001.zip › molecules-3057917-supplementary.pdf]

Supplementary material

For

Ruthenium *p*-cymene complexes incorporating substituted pyridine-quinoline based ligands. Synthesis, characterization, and cytotoxic properties.

Afroditi Kokkosi[1], Elpida Garofallidou[1], Nikolaos Zacharopoulos[1], Nikolaos Tsoureas[1], Konstantina Diamanti[2], Nikolaos Thomaidis[2], Antigoni Cheilari[3], Christina Machalia[4], Evangelia Emmanouilidou[4], Athanassios I. Philippopoulos[1*]

[1] Laboratory of Inorganic Chemistry, Department of Chemistry, National and Kapodistrian University of Athens, Panepistimiopolis Zografou 15771, Athens, Greece.

[2] Laboratory of Analytical Chemistry, Department of Chemistry, National and Kapodistrian University of Athens, Panepistimiopolis Zografou, 15771 Athens, Greece.

[3] Department of Pharmacognosy and Natural Products Chemistry, Faculty of Pharmacy, National and Kapodistrian University of Athens, Panepistimiopolis Zografou 15771, Athens, Greece.

[4] Laboratory of Biochemistry, Department of Chemistry, National and Kapodistrian University of Athens, Panepistimiopolis Zografou 15771

* Corresponding author: Prof. A. I. Philippopoulos. Tel.: +30-210-7274697; e-mail: atphilip@chem.uoa.gr

Table of Contents

Entry

**1) Figure S1**	ATR spectra of pqhyme, **1-Cl** and **1-PF$_6$**
**2) Figure S2**	^{1}H NMR of **1-Cl** in $CDCl_3$
**3) Figure S3**	^{13}C{^{1}H} NMR of **1-Cl** in $CDCl_3$
**4) Figure S4**	^{1}H NMR of **1-PF$_6$** in $CDCl_3$
**5) Figure S5**	^{13}C{^{1}H} NMR of **1-PF$_6$** in Me_2CO-d$_6$
**6) Figure S6**	^{1}H-^{1}H COSY of **1-PF$_6$** in Me_2CO-d$_6$
**7) Figure S7**	^{1}H NMR of **1-Cl** after 2 days in $CDCl_3$
**8) Figure S8**	X-ray structure of **1-Cl**•2$CHCl_3$•pqhyme
**9) Table S1**	Crystallographic data of **1-Cl**•2$CHCl_3$•pqhyme
**10) Figure S9**	Hydrogen bonding interactions in the unit cell of **1-Cl**•2$CHCl_3$•pqhyme
**11) Table S2**	A list of hydrogen bonding interactions within the crystal of **1-Cl**•2$CHCl_3$•pqhyme
**12) Figure S10**	Intermolecular non-classical C–H···π contacts in **1-PF$_6$**
**13) Figure S11**	ATR spectra of pqca, **2-Cl**
**14) Figure S12**	^{1}H NMR of **2-Cl** in DMSO-d$_6$
**15) Figure S13**	^{13}C{^{1}H} NMR of 2-**Cl** in DMSO-d$_6$
**16) Figure S14**	^{1}H-^{1}H COSY of 2-**Cl** in DMSO-d$_6$
**17) Figure S15**	^{1}H-^{13}C-HSQC of 2-**Cl** in DMSO-d$_6$
**18) Figure S16**	ATR spectra of pqcame, **3-Cl** and **3-PF$_6$**
**19) Figure S17**	^{1}H NMR of 3-**Cl** in $CDCl_3$
**20) Figure S18**	^{1}H-^{1}H COSY of **3-Cl** in $CDCl_3$
**21) Figure S19**	^{13}C{^{1}H} NMR of **3-Cl** in $CDCl_3$
**22) Figure S20**	^{1}H-^{13}C-HSQC of 3-**Cl**
**23) Figure S21**	^{1}H NMR of **3-PF$_6$** in Me_2CO-d$_6$
**26) Figure S22**	^{13}C{^{1}H} NMR of **3-PF$_6$** in Me_2CO-d$_6$
**27) Figure S23**	^{1}H-^{1}H COSY of **3-PF$_6$** in Me_2CO-d$_6$

28) **Figure S24** Intramolecular and intermolecular contacts in **3-PF**$_6$

29) **Figure S25** UV-Vis spectrum of **1-Cl**, in DMSO (not dry, 10^{-4} M), over 1h.

30) **Figure S26** UV-Vis spectrum of **2-Cl**, in DMSO (not dry, 10^{-4} M), over time

31) **Figure S27** UV-Vis of **3-Cl** in H_2O over time (7.8 ×10^{-5} M)

32) **Figure S28** Stability of 1-**Cl** checked by ^{1}H NMR spectroscopy in DMSO-d_6 over time

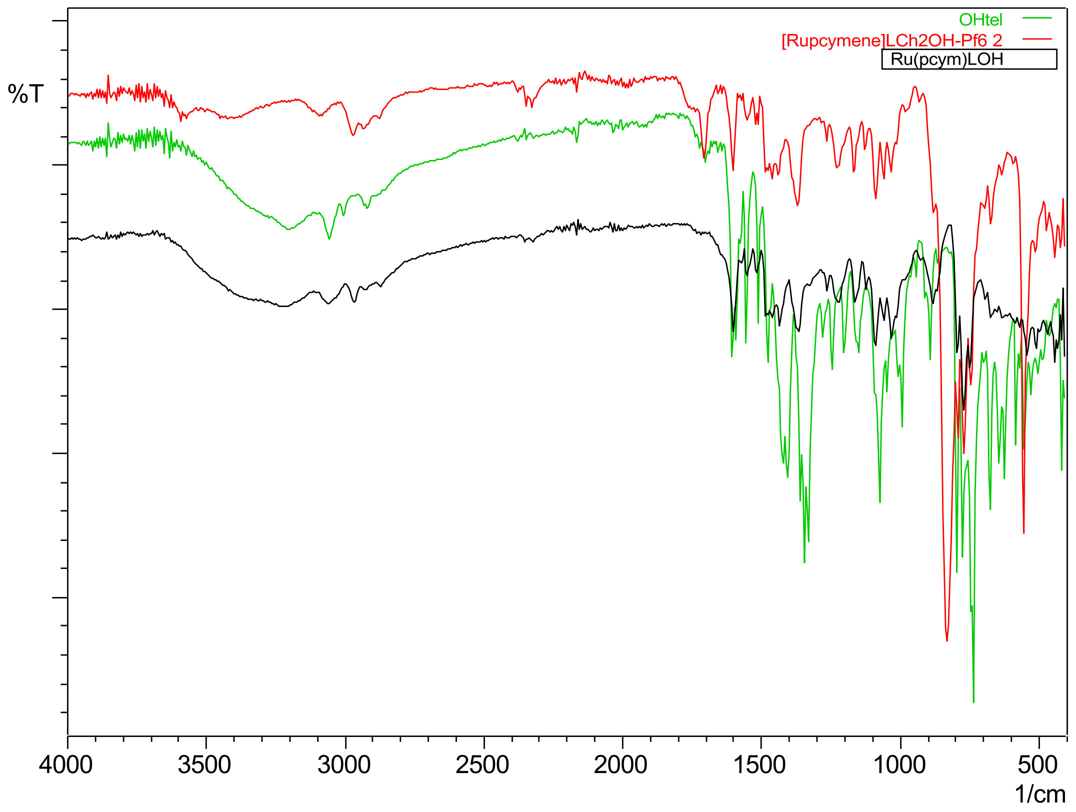

Fig.S1 ATR spectrum of pqhyme, **1-Cl** and **1-PF**$_6$

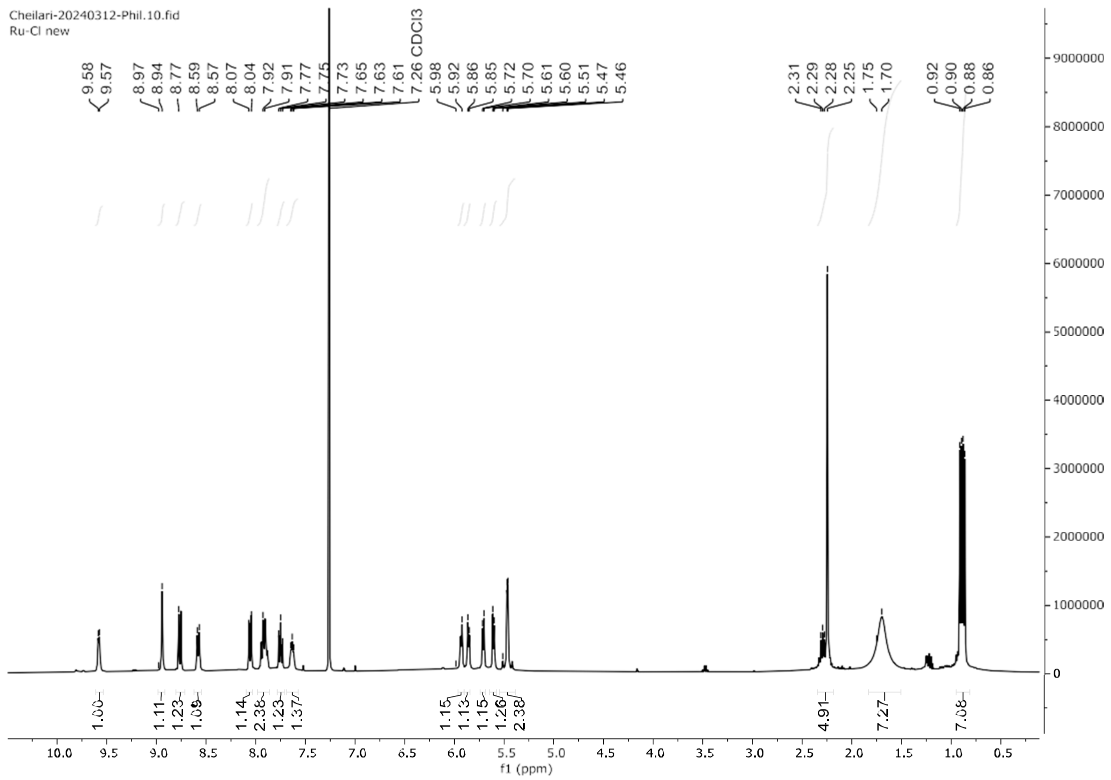

Fig.S2 ^{1}H NMR of **1-Cl** in $CDCl_3$

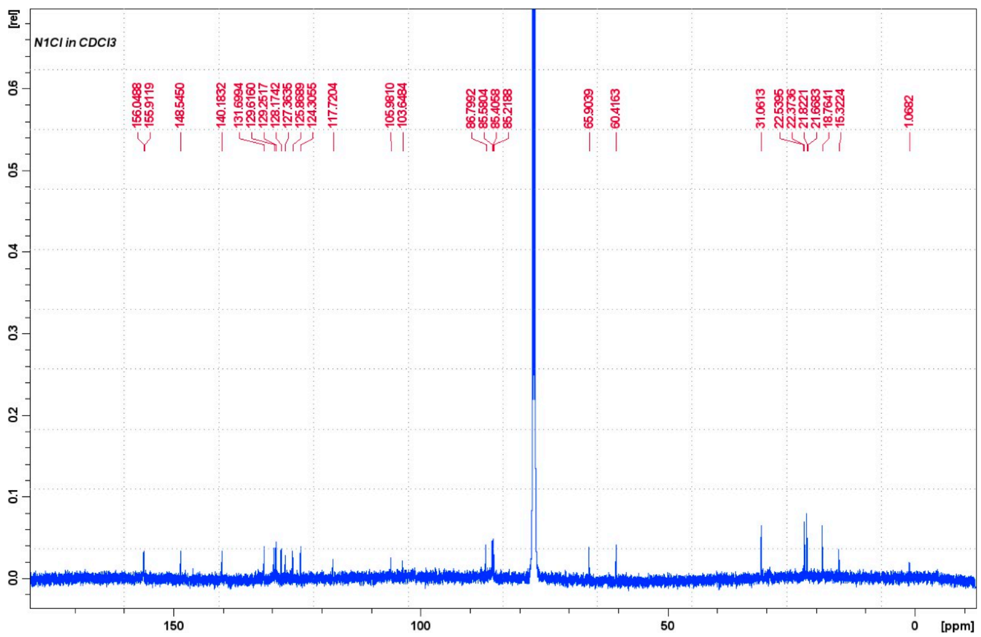

Fig.S3 $^{13}C\{^{1}H\}$ NMR of **1-Cl** in $CDCl_3$

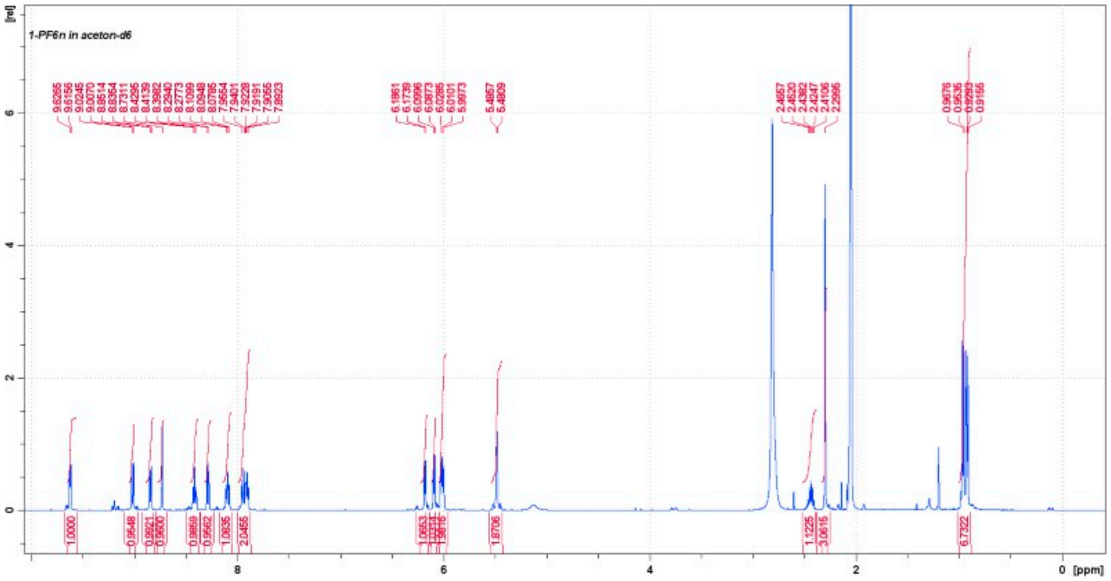

Fig.S4 ^{1}H NMR of **1-PF$_6$** in Me$_2$CO-d$_6$

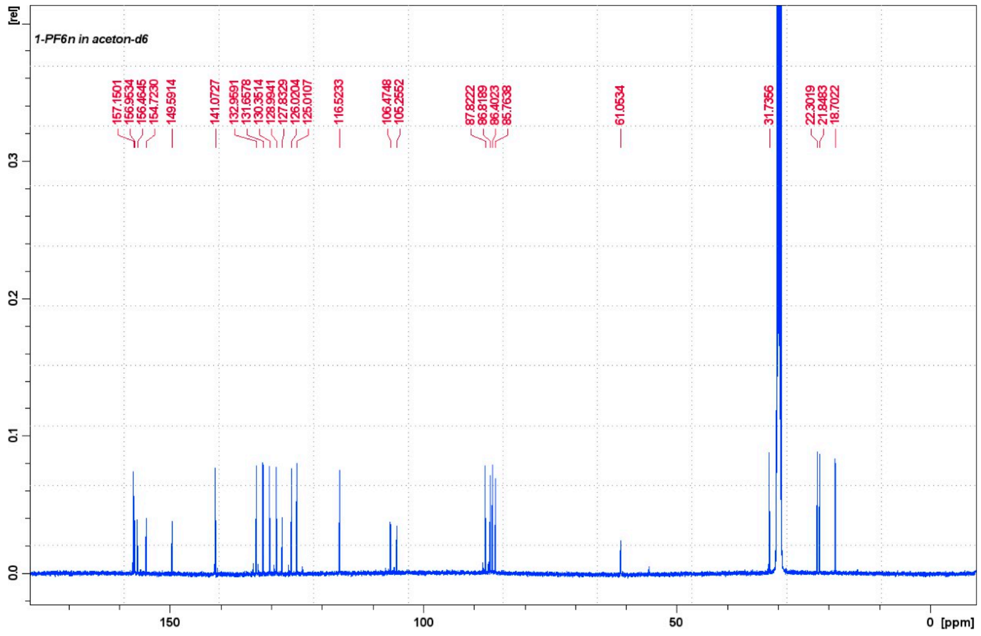

Fig. S5. $^{13}C\{^1H\}$ NMR of **1-PF$_6$** in Me$_2$CO-d$_6$

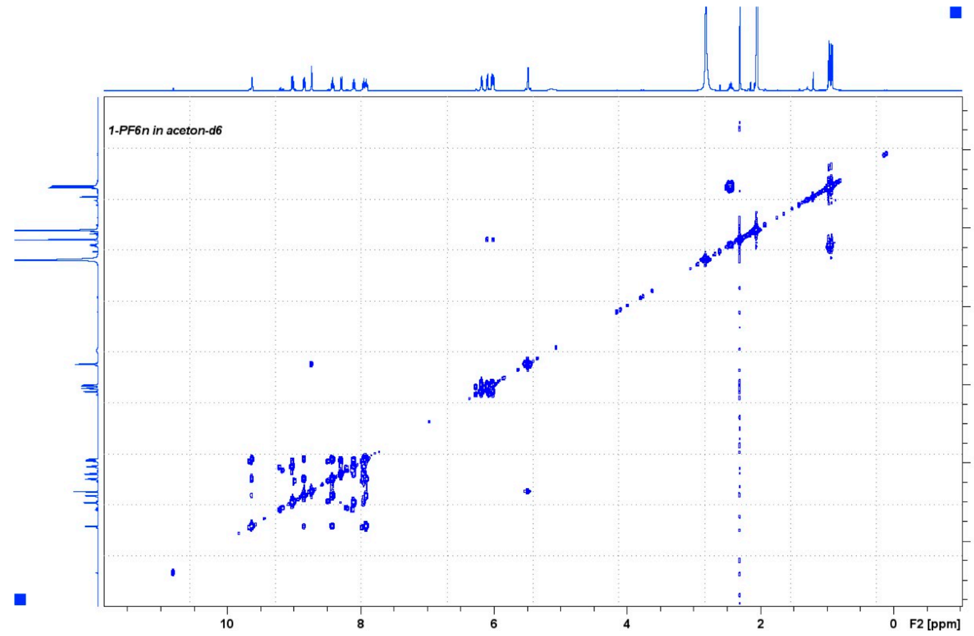

Fig. S6. ^{1}H-^{1}H COSY of **1-PF$_6$** in Me$_2$CO-d$_6$

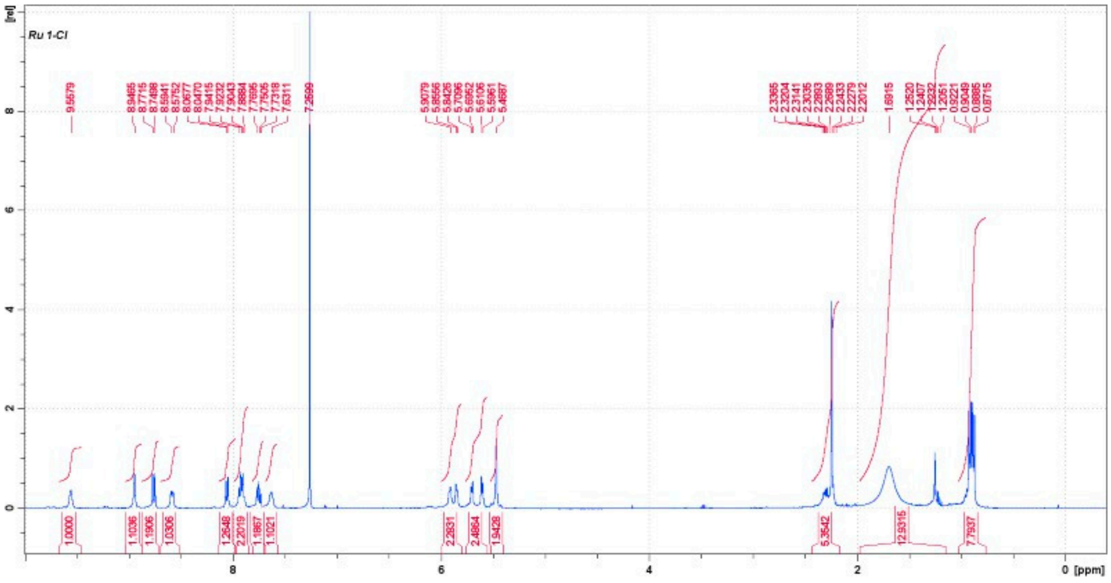

Fig. S7. ^{1}H NMR of **1-Cl** after 2 days in CDCl$_3$

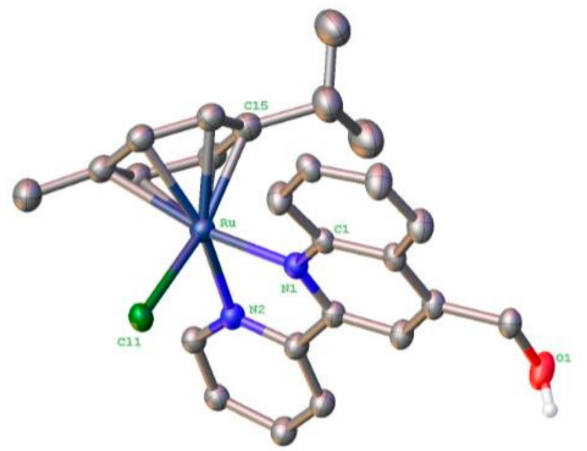

Fig. S8. X-ray structure of **1-Cl**•2$CHCl_3$•pqhyme

Table S1. Crystallographic data of **1-Cl**•2$CHCl_3$•pqhyme

Identification code	Phili90, KA1 // GXraymo_6841_twin1
Crystal Habitus	clear yellow plate
Device Type	Bruker D8 Venture
Empirical formula	$C_{42}H_{40}Cl_8N_4O_2Ru$
Moiety formula	C25 H26 Cl N2 O Ru, Cl, 2(C H Cl3), C15 H12 N2 O
Formula weight	1017.45
Temperature/K	100.15
Crystal system	monoclinic
Space group	Ia
a/Å	17.1871(3)
b/Å	15.5639(2)
c/Å	33.5662(6)
α/°	90
β/°	103.159(2)
γ/°	90
Volume/Å^3	8743.1(3)
Z	8
ρ_{calc}g/cm^3	1.546
μ/mm^{-1}	0.889
F(000)	4128.0
Crystal size/mm^3	0.18 × 0.16 × 0.04
Absorption correction	empirical
Tmin; Tmax	0.460293; 0.745553
Radiation	MoKα (λ = 0.71073)
2Θ range for data collection/°	3.574 to 55.998°
Completeness to theta	1.000
Index ranges	-22 ≤ h ≤ 22, -20 ≤ k ≤ 20, -44 ≤ l ≤ 44
Reflections collected	118525
Independent reflections	21097 [R_{int} = 0.1147, R_{sigma} = 0.0512]

Data/restraints/parameters	21097/1010/1038
Goodness-of-fit on F^2	1.072
Final R indexes [I>=2σ (I)]	R_1 = 0.1159, wR_2 = 0.2754
Final R indexes [all data]	R_1 = 0.1239, wR_2 = 0.2815
Largest diff. peak/hole / e Å^{-3}	4.52/-1.82
Flack parameter	0.29(10)

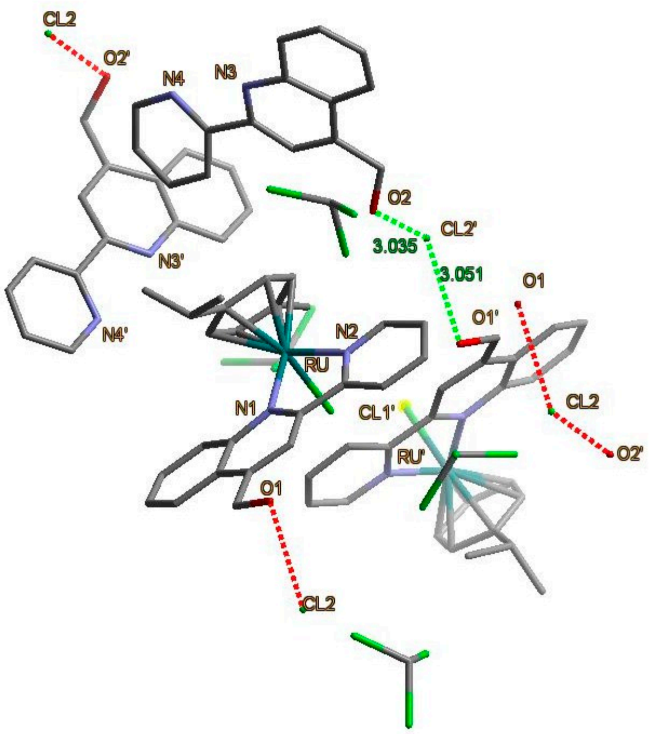

Fig. S9. Hydrogen bonding interactions in the unit cell of **1-Cl**•2$CHCl_3$•pqhyme

Table S2. A list of hydrogen bonding interactions within the crystal of **1-Cl**•2$CHCl_3$•pqhyme

D	H	A	d(D-H)/Å	d(H-A)/Å	d(D-A)/Å	D-H-A/°
O1	H1	Cl2[1]	0.84	2.29	3.099(17)	160.7
C6	H6	Cl2'[2]	0.95	2.84	3.54(2)	131.4
C11	H11	Cl1'[3]	0.95	2.69	3.57(2)	155.7
C14	H14	Cl2'	0.95	2.74	3.627(19)	155.6
C14	H14	O2	0.95	2.62	3.09(2)	111.2
O1'	H1'	Cl2'	0.84	2.25	3.051(15)	158.6
C6'	H6'	Cl2[4]	0.95	2.92	3.592(18)	128.9
C9'	H9'A	Cl6	0.99	2.97	3.45(2)	111.1

C11'	H11'	Cl1	0.95	2.68	3.541(19)	150.4
C14'	H14'	Cl2^{2}	0.95	2.70	3.618(18)	161.9
C40'	H40'	N3^{5}	1.00	2.31	3.25(3)	156.0
C41	H41	Cl1	1.00	2.95	3.62(2)	124.8
C41	H41	O1'	1.00	2.34	3.15(3)	137.0
C41'	H41'	O1^{4}	1.00	2.40	3.16(3)	132.3
C41'	H41'	Cl1'	1.00	2.84	3.58(2)	131.5
C34	H34A	N3'^{6}	0.99	2.61	3.37(3)	133.7
C34	H34B	Cl7'^{6}	0.99	2.84	3.81(2)	166.5
O2'	H2'	Cl2^{7}	0.84	2.30	3.099(16)	159.5

^{1}-1/2+X,2-Y,+Z; ^{2}-1/2+X,1-Y,+Z; ^{3}+X,1+Y,+Z; ^{4}+X,-1+Y,+Z; ^{5}+X,3/2-Y,1/2+Z; ^{6}1/2+X,1-Y,+Z; ^{7}-1/2+X,-1/2+Y,-1/2+Z

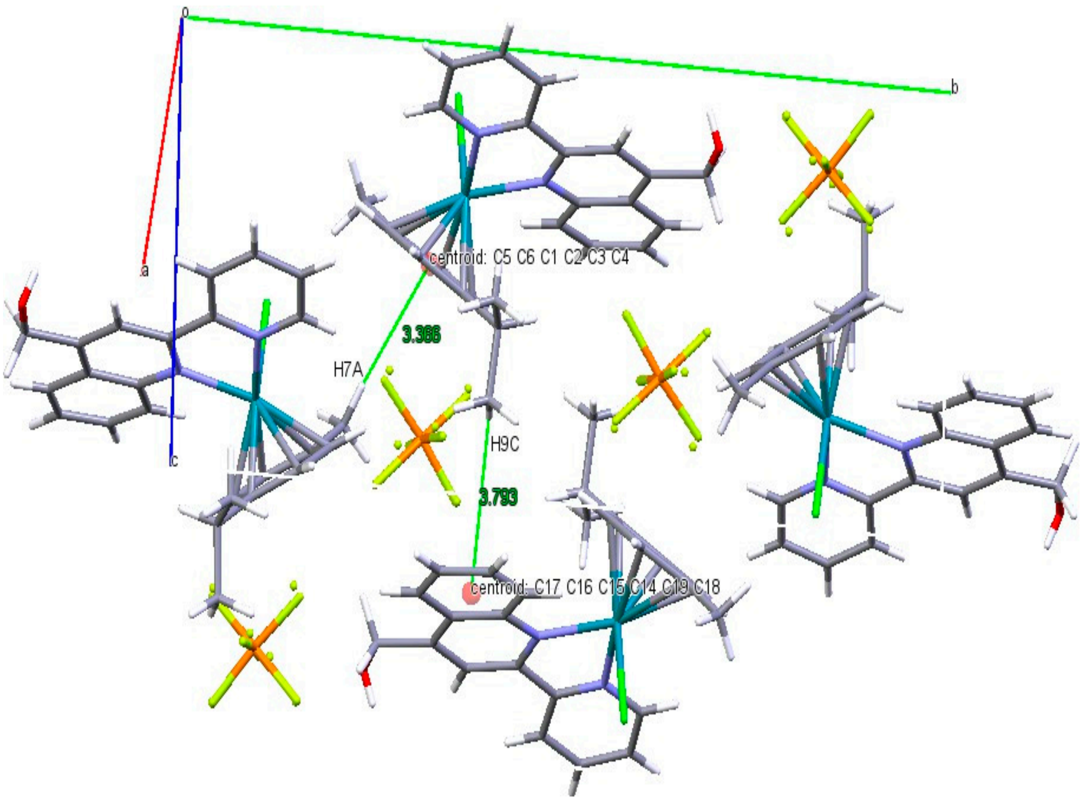

Figure S10. Intramolecular non-classical C–H···π contacts in **1-PF$_6$**

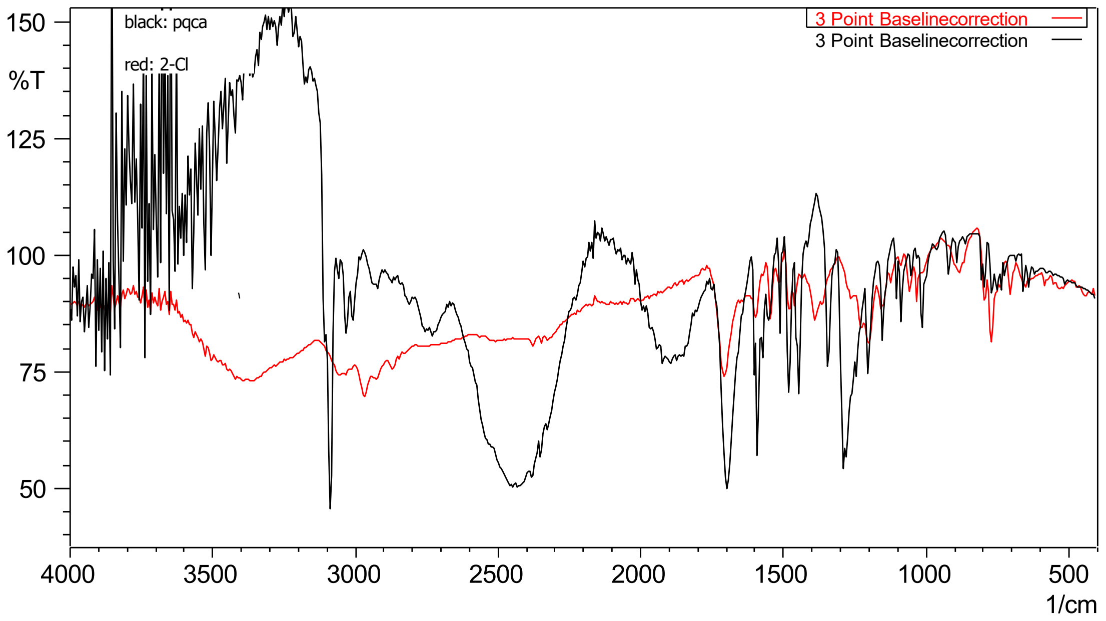

Figure S11. ATR spectra of pqca and complex **2-Cl**.

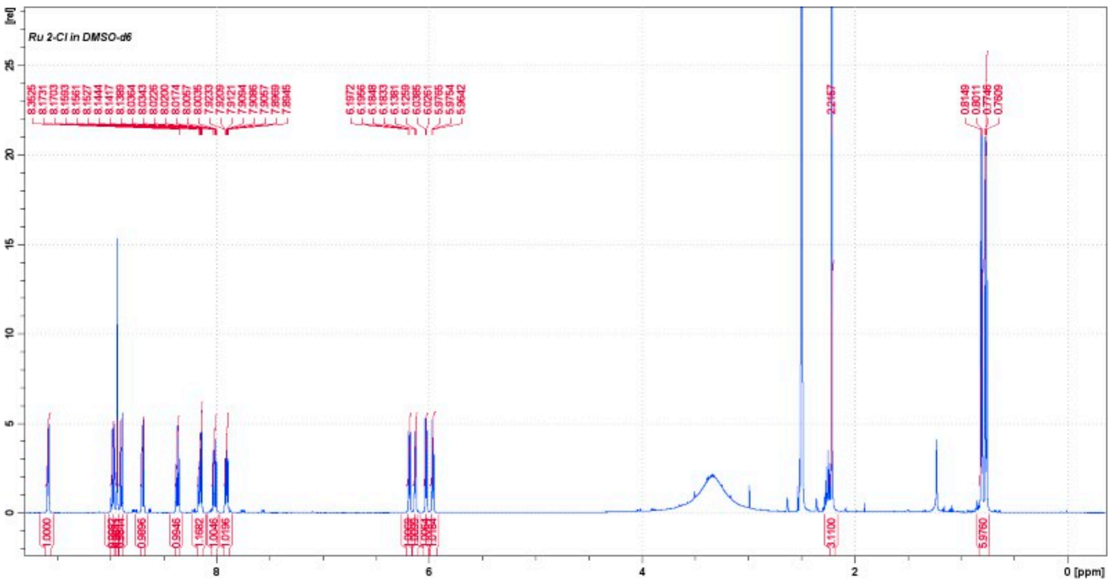

Figure S12. ^{1}H NMR of **2-Cl** in DMSO-d$_6$

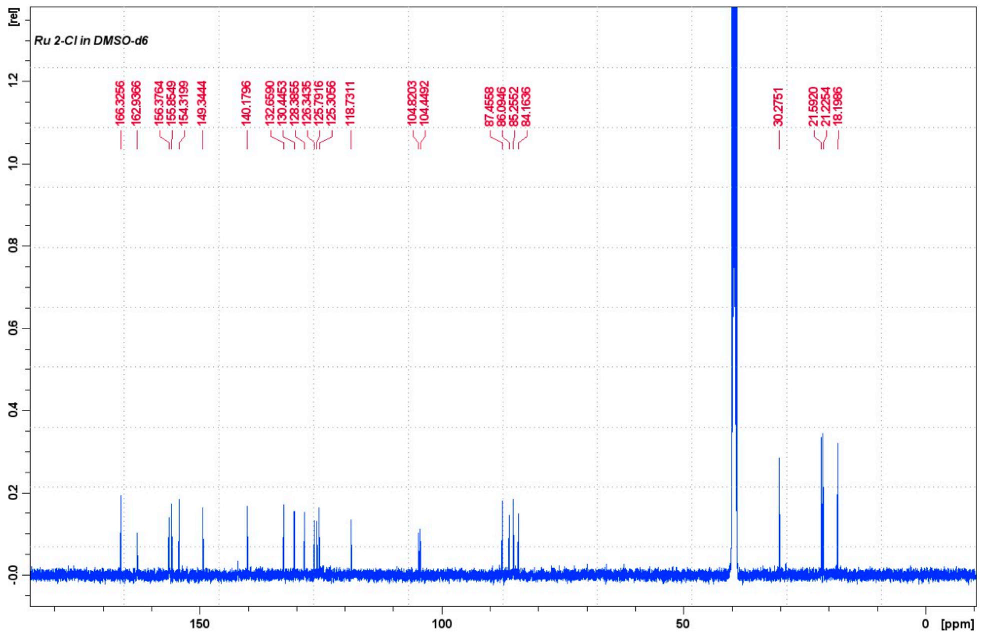

Figure S13. $^{13}C\{^{1}H\}$ NMR of 2-**Cl** in DMSO-d$_6$

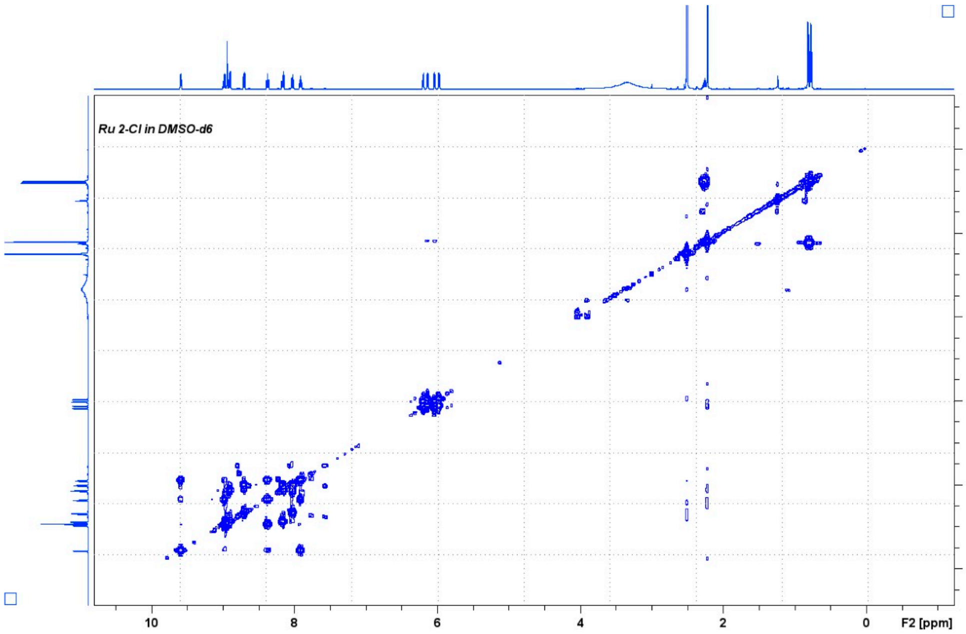

Figure S14. ^{1}H-^{1}H COSY of 2-**Cl** in DMSO-d$_6$

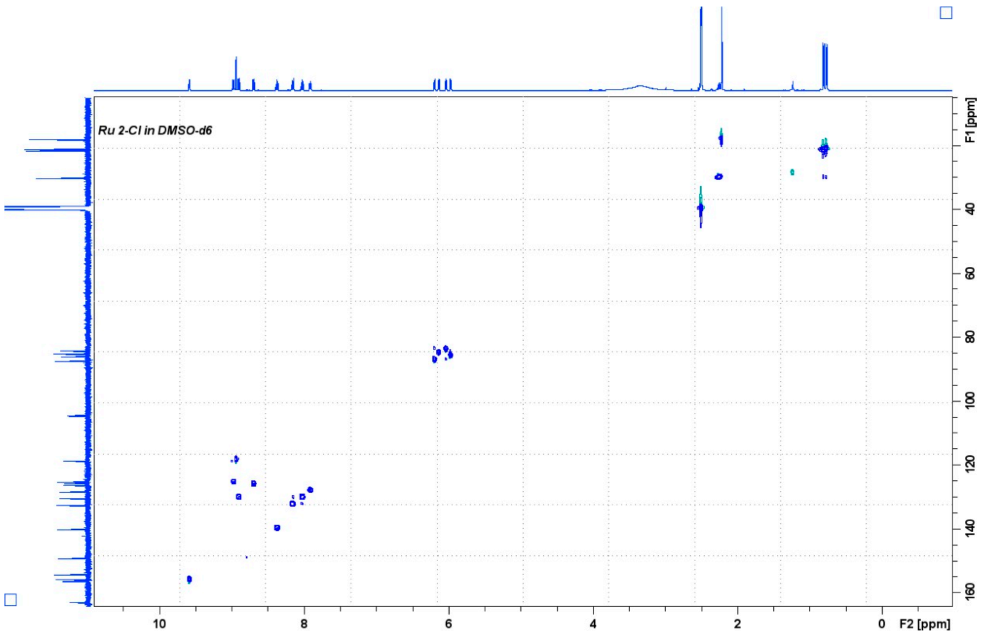

Figure S15. ^{1}H-^{13}C-HSQC of **2-Cl** in DMSO-d_6

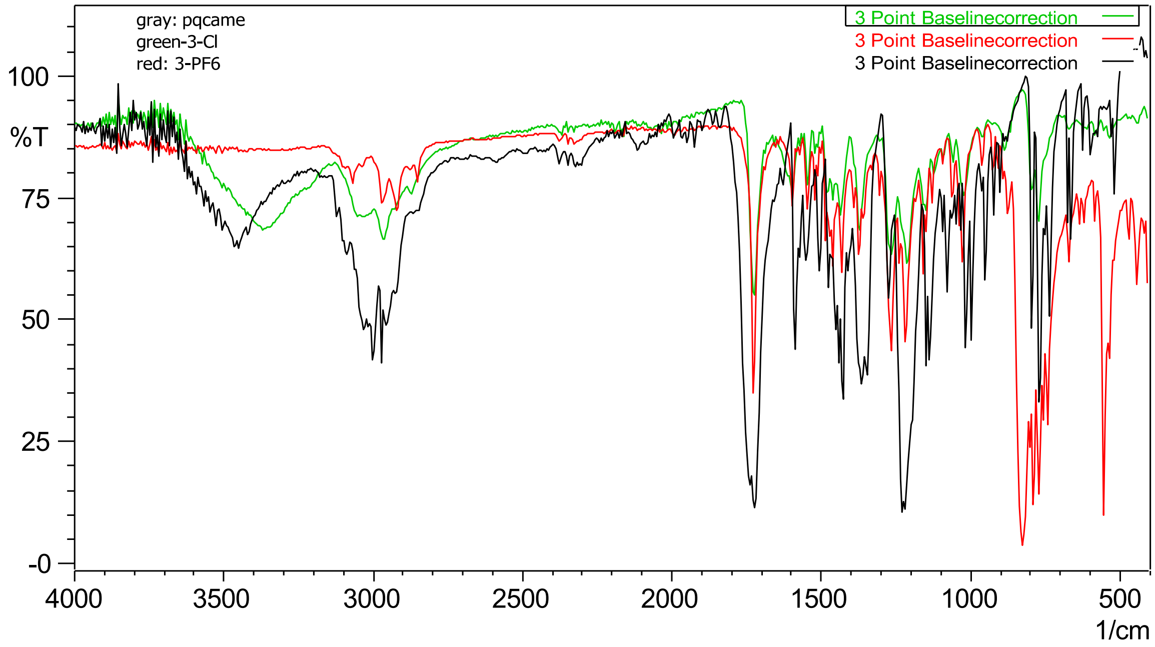

Figure S16: ATR spectra of pqcame, **3-Cl** and **3-PF$_6$**.

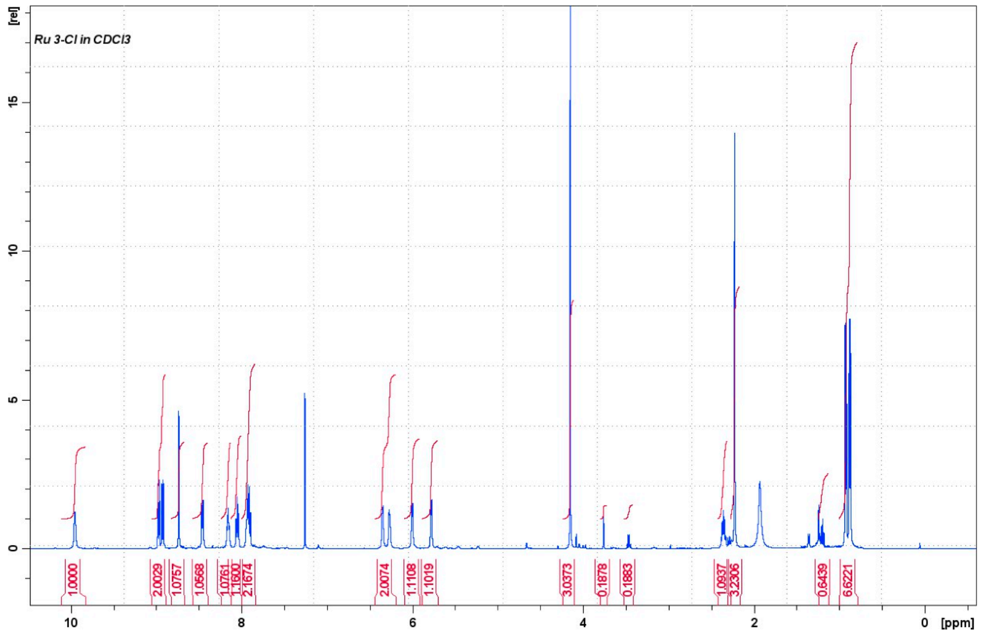

Figure S17. ^{1}H NMR of **3-Cl** in $CDCl_3$

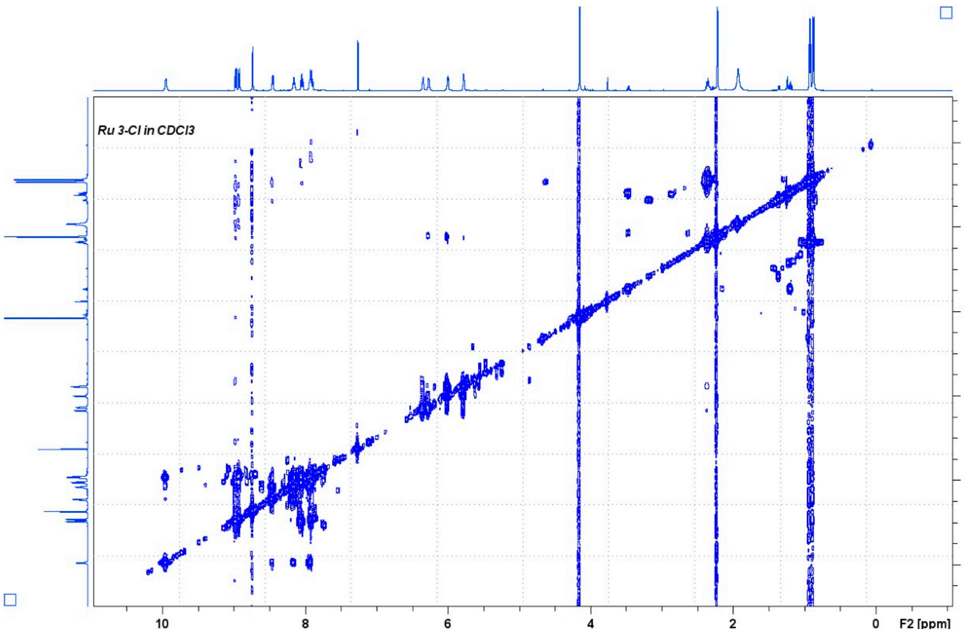

Figure S18. ^{1}H-^{1}H COSY of **3-Cl** in $CDCl_3$

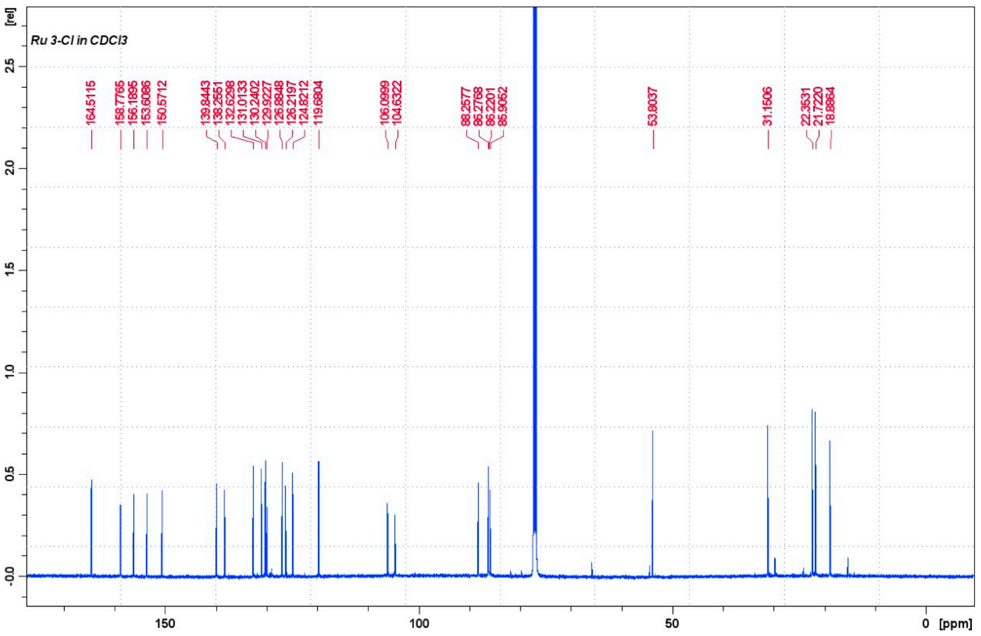

Figure S19. $^{13}C\{^{1}H\}$ NMR of **3-Cl** in $CDCl_3$

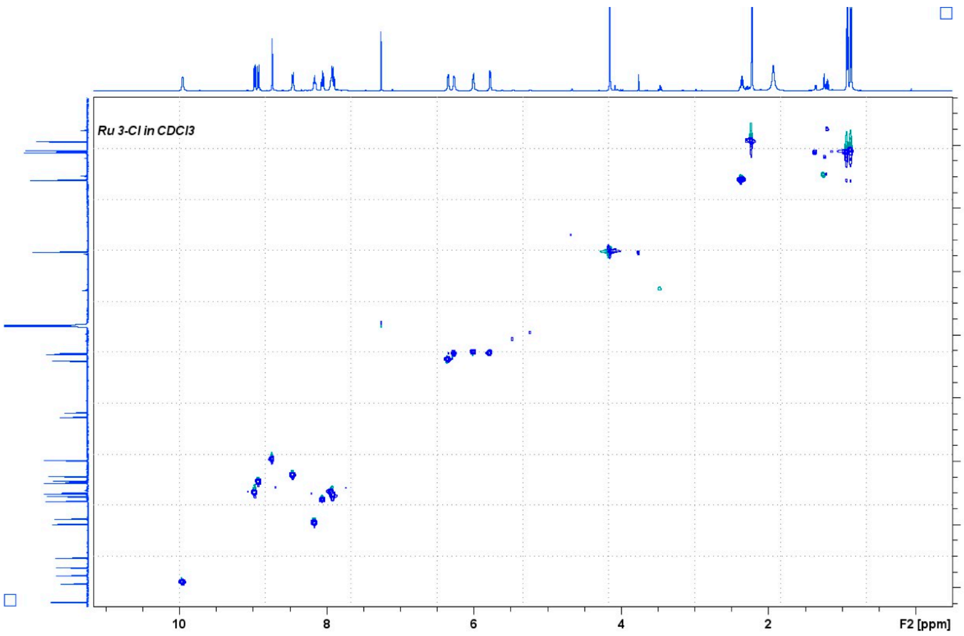

Figure S20. ^{1}H-^{13}C-HSQC of **3-Cl** in $CDCl_3$

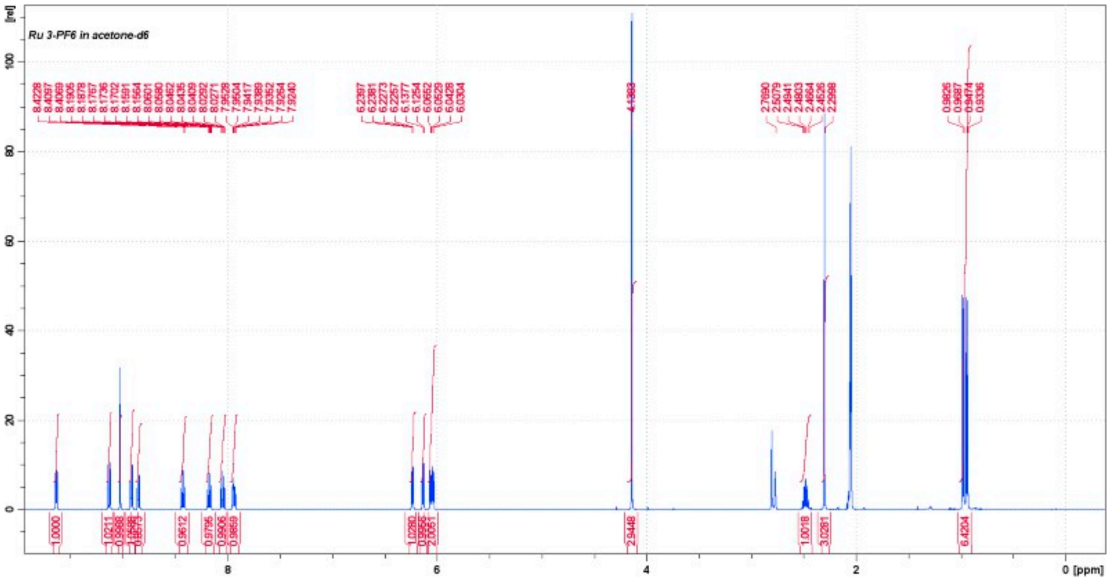

Figure S21. ^{1}H NMR of **3-PF$_6$** in Me$_2$CO-d$_6$

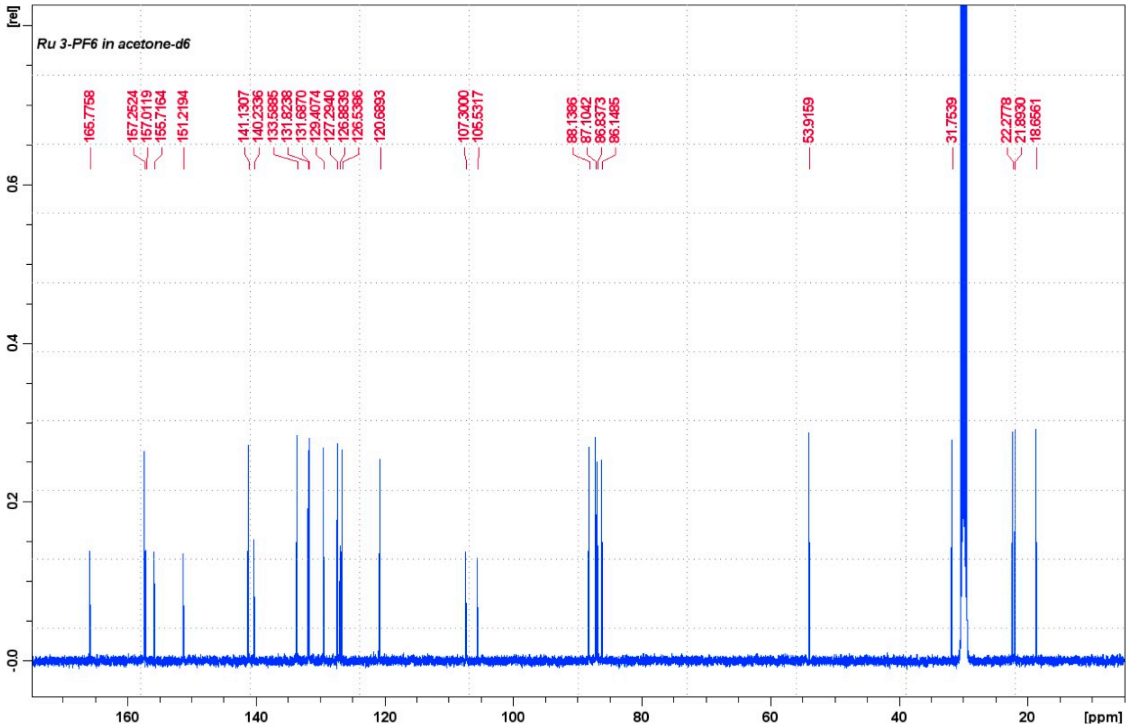

Figure S22. ^{13}C{^{1}H} NMR of **3-PF$_6$** in Me$_2$CO-d$_6$

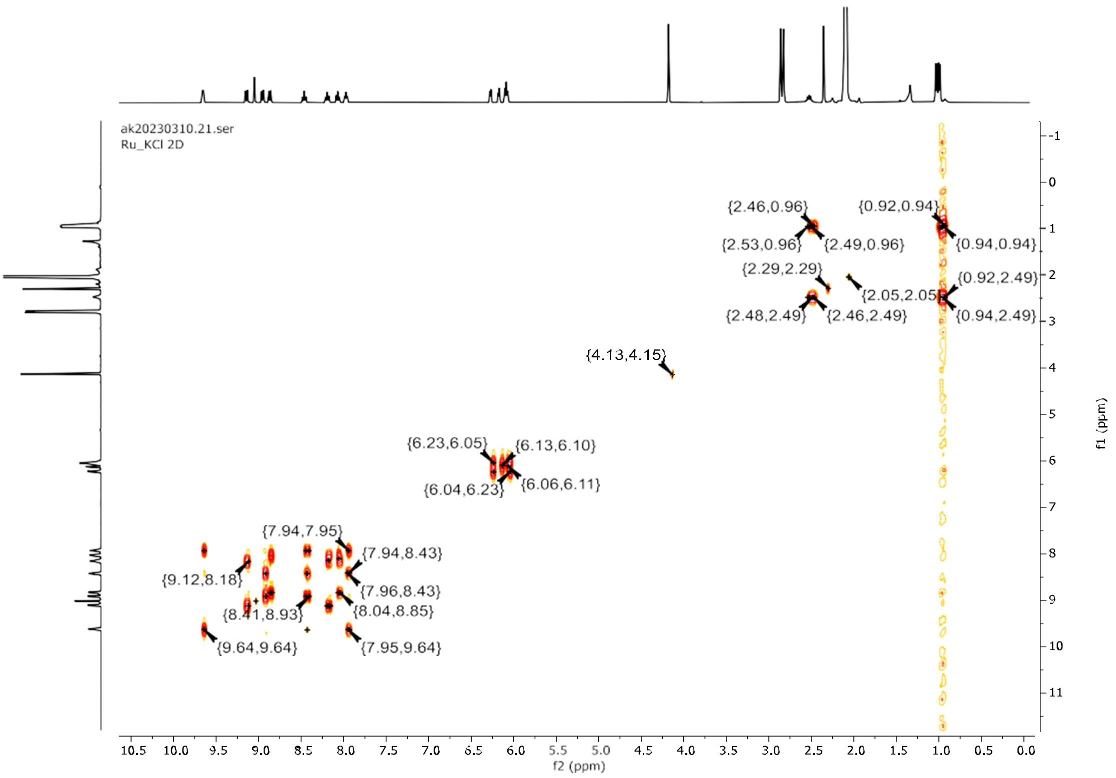

Figure S23. ^{1}H-^{1}H COSY of **3-PF$_6$** in Me$_2$CO-d$_6$

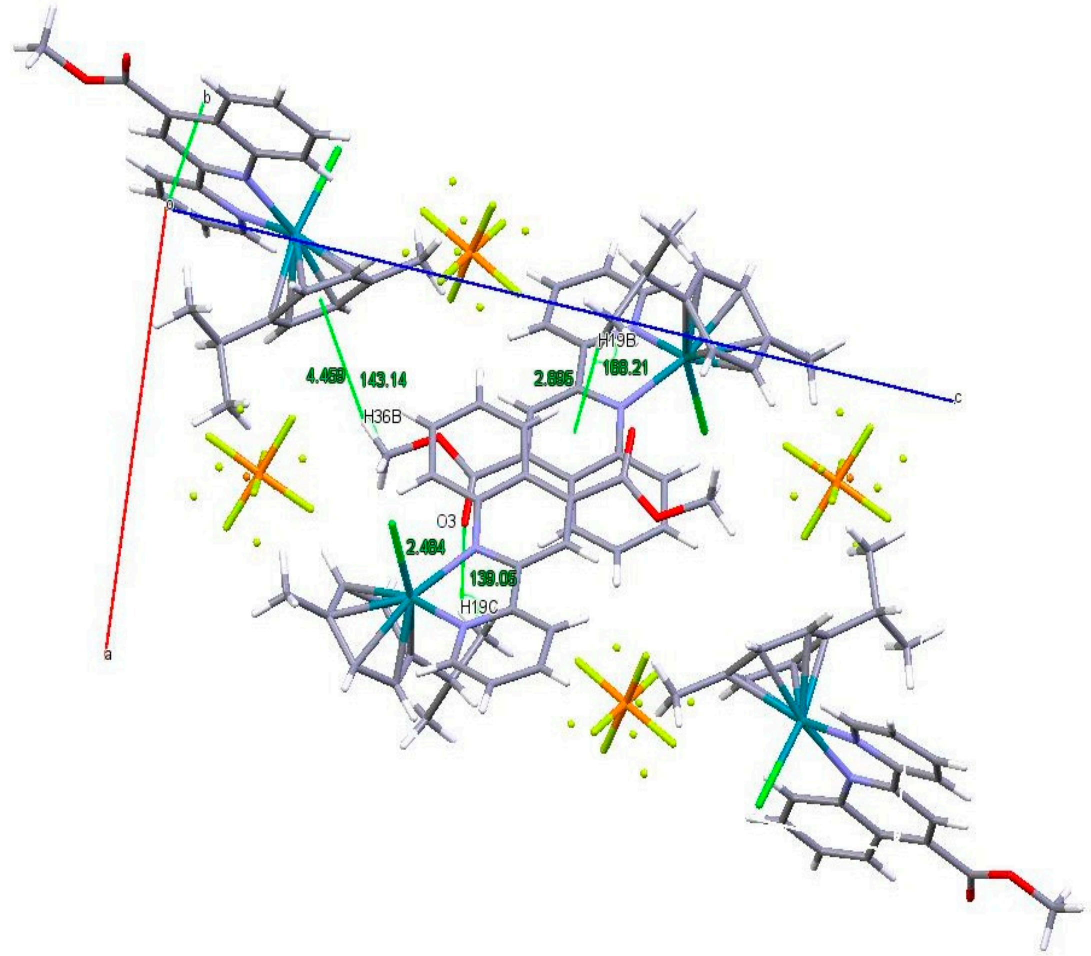

Figure S24. Intramolecular and intermolecular contacts in **3-PF**$_6$.

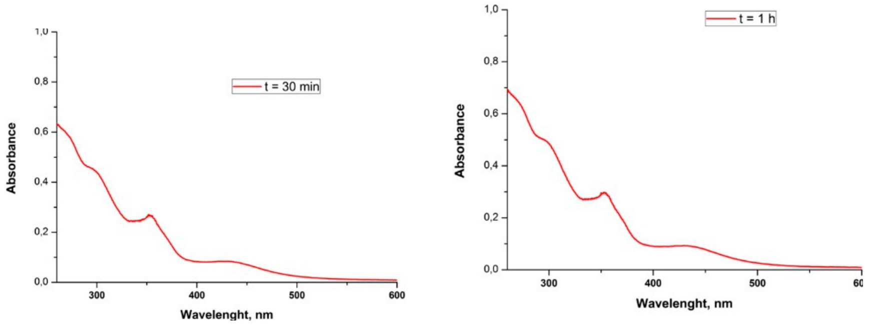

Figure S25. UV-Vis spectrum of **1-Cl**, in DMSO (not dry, 10^{-4} M), over 1h.

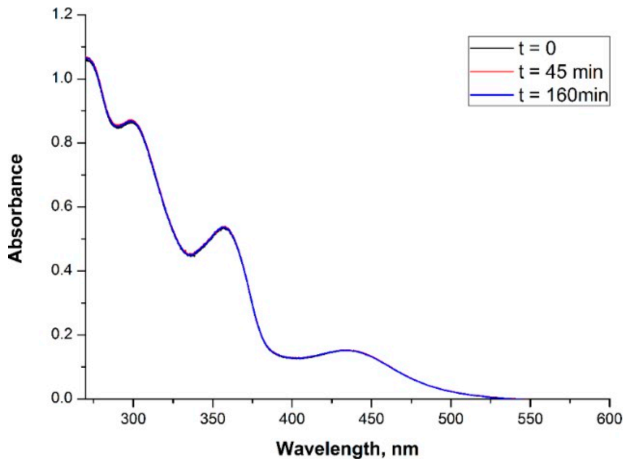

Figure S26. UV-Vis spectrum of **2-Cl**, in DMSO (not dry, 10^{-4} M), over time.

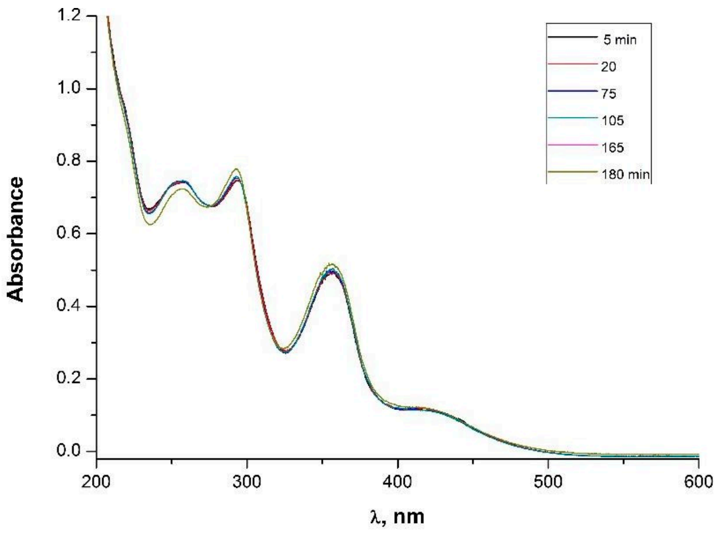

Figure S27. UV-Vis of **3-Cl** in H_2O over time (7.8 ×10^{-5} M)

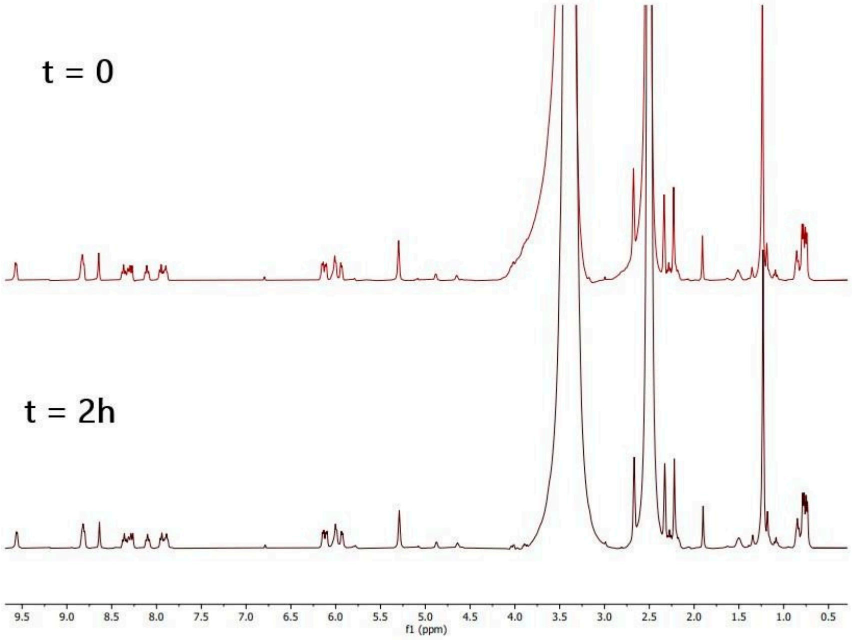

Figure S28. Stability of **1-Cl**, checked by ^{1}H NMR, in DMSO-d_6 over time (2 h).
